# Circular RNA-ZBTB44 regulates the development of choroidal neovascularization

**DOI:** 10.7150/thno.39488

**Published:** 2020-02-10

**Authors:** Rong-mei Zhou, Lian-jun Shi, Kun Shan, Ya-nan Sun, Shan-shan Wang, Shu-jie Zhang, Xiu-miao Li, Qin Jiang, Biao Yan, Chen Zhao

**Affiliations:** 1 Eye Institute, Eye & ENT Hospital, Shanghai Medical College, Fudan University, Shanghai, China; 2 The Fourth School of Clinical Medicine, Nanjing Medical University, Nanjing, China; 3 Eye Hospital, Nanjing Medical University, Nanjing, China; 4 NHC Key Laboratory of Myopia (Fudan University), Key Laboratory of Myopia, Chinese Academy of Medical Sciences, and Shanghai Key Laboratory of Visual Impairment and Restoration (Fudan University), Shanghai, China; 5 Department of Ophthalmology, The First Affiliated Hospital of Nanjing Medical University, State Key Laboratory of Reproductive Medicine, Nanjing, China; 6 State Key Laboratory of Ophthalmology, Zhongshan Ophthalmic Center, Sun Yat-sen University, Guangzhou, China

**Keywords:** choroidal neovascularization, circular RNA, cZBTB44, miR-578 sponge

## Abstract

**Rationale**: Choroidal neovascularization (CNV) is a major cause of severe vision loss and occurs in many ocular diseases, especially neovascular age-related macular degeneration (nAMD). Circular RNAs (circRNAs) are emerging as a new class of endogenous noncoding RNAs, which have been implicated in the regulation of endothelial cell dysfunction in diabetes mellitus and cancer. In this study, we aimed to determine the role of circRNA-ZBTB44 (cZBTB44) in the pathogenesis of CNV.

**Methods**: Quantitative polymerase chain reaction was conducted to detect cZBTB44 expression pattern during CNV development. Isolectin B4 staining, hematoxylin and eosin (HE) staining, and choroidal sprouting assay *ex vivo* were conducted to evaluate the role of cZBTB44 in the development of CNV. Endothelial cell proliferation, migration and tube formation assays were conducted to determine the role of cZBTB44 in angiogenic effect *in vitro*. Bioinformatics analysis, RNA immunoprecipitation assay, luciferase assay, and *in vitro* studies were conducted to investigate the mechanism of cZBTB44-mediated CNV development.

**Results**: cZBTB44 expression was significantly up-regulated in a laser-induced CNV mouse model *in vivo* and in endothelial cells upon hypoxia stress *in vitro*. cZBTB44 silencing retarded CNV development, while overexpression of cZBTB44 showed the opposite effects. The role of cZBTB44 in CNV development was confirmed in choroidal sprouting assay *ex vivo*. cZBTB44 silencing reduced endothelial cell viability, proliferation, migration and tube formation *in vitro*. cZBTB44 acted as miR-578 sponge to sequester and inhibit miR-578 activity, which led to increased expression of vascular endothelial growth factor A (VEGFA) and vascular cell adhesion molecule-1 (VCAM1). Overexpression of miR-578 mimicked cZBTB44 silencing-mediated anti-angiogenic effects *in vivo* and *in vitro*. Furthermore, dysregulated cZBTB44 expression was detected in the clinical samples of nAMD patients.

**Conclusions**: This study provided novel insights into the molecular pathogenesis of CNV. The cZBTB44-miR-578-VEGFA/VCAM1 axis might be a potential source of novel therapeutic targets for neovascularization-related diseases.

## Introduction

Choroidal neovascularization (CNV) is the major pathological manifestation of multiple blinding eye diseases such as neovascular age-related macular degeneration (nAMD), pathologic myopia as well as ocular fundus trauma 1, 2. It refers to the outgrowth of new blood vessels from the choroidal circulation, which penetrate through Bruch's membrane to retinal pigment epithelium (RPE) or hide beneath the retina 3, 4. CNV usually leads to edema, exudation, hemorrhage and outer retinal dysfunction, with vision loss as a direct result 5. Currently, the therapeutic modalities for CNV include laser photocoagulation, vitrectomy surgery and anti-vascular endothelial growth factor (VEGF) 6. However, laser photocoagulation and vitrectomy surgery have limited efficacy and damage retinal parenchyma and vasculature. Frequent intravitreal injections of anti-VEGF biologics may cause endophthalmitis and retinal tears 7, 8. Therefore, in-depth study about the pathogenesis of CNV and search for novel molecular targets are still needed to improve the anti-angiogenic treatment of CNV.

Circular RNAs (circRNAs) are a novel class of widely expressed noncoding RNAs that have covalently closed loop structures. Because circRNAs have no 5'-cap and no 3'-polyadenylated tail structure, they show highly stable comparing to their linear counterparts. In addition, a large number of circRNAs are reported to be conserved, cell type-specific or developmental stage-specific, supporting the concept of circRNAs as functional molecules 9-12. They have emerged as critical players in regulating gene expression by serving as microRNA (miRNA) sponges and interaction with RNA-binding protein as well as nuclear transcriptional regulators 11, 13. Increasing studies have identified that aberrant circRNA expressions were detected in vascular diseases, neurodegenerative diseases and cancers 14-17. CNV is one of the common pathogenesis of vascular diseases in the posterior of the eye. However, the role of circRNAs in CNV is still unknown.

In this study, we characterized the expression pattern of circular RNA-ZBTB44 and investigated its role in the pathogenesis of CNV. cZBTB44 (has_circ_0002484) is located at chro11: 130130750-130131824. It displays high conservation between mouse genome and human genome 18, 19. We revealed that cZBTB44 was significantly up-regulated in the choroid of laser-induced CNV mice and in endothelial cells upon hypoxia stress. cZBTB44 knockdown attenuated laser-induced CNV formation *in vivo* and *ex vivo*, and exhibited anti-angiogenic activity in endothelial cells *in vitro*. The intervention of cZBTB44 can become a promising therapeutic strategy for neovascularization-related diseases.

## Methods

### Ethics statement

All animal protocols were approved by the Animal Care and Use Committee of Eye & ENT Hospital and Nanjing Medical University. All mice were handled in compliance with the Association for Research in Vision and Ophthalmology Statement for the Use of Animals in Ophthalmic and Vision Research. The clinical specimens were handled in accordance with the Declaration of Helsinki. All patients gave the informed consent before inclusion.

### Cell culture and transfection

RF/6A cells were cultured in Iscove's Modified Dulbecco's Medium (*IMDM, Gibco*) containing 10% heat-inactivated fetal bovine serum (FBS) (Gibco, Cat. 10099141), 10 IU/mL penicillin, 10 *μ*g/mL streptomycin (Gibco, Cat. 15140122) at 37 °C and 5% CO_2_. Small interfering RNAs (siRNAs) were designed and synthesized by RiboBio Technology (Guangzhou, China). Lipofectamine 3000 (Invitrogen, Cat. L3000015) was used to introduce siRNAs into RF/6A cells according to the manufacturer's protocol. The siRNA target sequence was shown as follows: cZBTB44 siRNA1: 5'- AATTCTGCAAAGTGACAGATT -3'; cZBTB44 siRNA2: 5'- GCAAAGTGACAGATTGCAGTA-3'; cZBTB44 siRNA3: 5'- GATCAATTCTGCAAAGTGACA-3'.

### Laser-induced CNV in mice

Four laser burns surrounding the optic disc were induced by a green Argon laser pulse (Micron IV, Phoenix Research Laboratories, Pleasanton, CA, USA) with duration of 100 ms and power of 100 mW in 6-8 weeks old mice. Disruption of Bruch's membrane was confirmed by white bubble formation. Mice were locally administrated with adeno-associated virus vectors containing cZBTB44-small hairpin (shRNA) or scrambled shRNA by intravitreal injection. Two weeks after laser treatment, eyes were enucleated and fixed in 4% paraformaldehyde for 30 min at room temperature. The flat-mounts were penetrated with 1% Triton X-100 PBS for 40 min at room temperature and stained with fluorescein coagulated Isolectin B4 (1:500, Alexa Fluor 594, I21413, Molecular Probes, Life Technologies) at 4 °C overnight. The flatmounts were washed with PBS and observed using a fluorescence microscope (Nikon, Tokyo, Japan).

### Choroidal sprouting assay *ex vivo*

Six-week-old C57BL/6 mice were killed and eyes were immediately enucleated and kept in ice-cold DMEM. The choroidal explants containing RPE/choroid/sclera complex from the peripheral area were isolated and cut into approximately 1 × 1 mm^2^ pieces. The choroidal explants were immediately embedded in 40 μL growth factor-reduced Matrigel (BD Biosciences, Cat. 354230) in 24-well plates (day 0). The explants were grown in 500 μL DMEM high glucose with 10% FBS (Gibco, Cat. 10099141) and 1% Penicillin/Streptomycin (Gibco, Cat. 15140122) at 37 °C with 5% CO_2_. At day 1, day 3 and day 5, images were taken under 25 × magnification respectively. The sprouting area was quantified using ImageJ software.

### RNase R treatment

For RNase R treatment, approximately 2 μg of total RNA was incubated with or without 3U μg^-1^ of RNase R for 30 min at 37 °C. The resulting RNA was purified using the RNeasy MinElute cleaning Kit (Qiagen).

### Plasmids construction and transfection

For the construction of the cZBTB44 over-expression vector, cZBTB44 cDNA was synthesized and cloned into a pcDNA3.1 vector (RiboBio, Guangzhou, China) between *XhoI* and *BamHI* restriction sites. Transfection was carried out using Lipofectamine 3000 (Invitrogen) according to the manufacturer's instructions.

### RNA immunoprecipitation assay (RIP)

RIP was conducted in RF/6A cells 48 h post-transfection with miR-578 mimics or miR-NC, using Magna RIPTM RNA-binding protein immunoprecipitation kit (Millipore, Billerica, MA). RF/6A cells were washed with ice-cold PBS and lysed in complete RNA lysis buffer. Then cell lysates were incubated with the primary antibody at 4 °C for 3 h (Ago2 or IgG). Samples were incubated with Proteinase K and then immunoprecipitated RNA was isolated. Extracted RNAs were analyzed by qRT-PCRs to identify the presence of cZBTB44.

### Biotin-coupled miRNA capture

The 3′ end biotinylated miR-578 or control mimic RNA (RiboBio) was transfected into RF/6A cells for 24 h at the concentration of 30 nM. The biotin-conjugated RNA complex was pulled down by incubating the cell lysates with streptavidin-coated magnetic beads (Life Technologies). The amount of cZBTB44 in the bound portion was detected by qRT-PCR assays.

### Dual luciferase activity assay

The 3′-UTR or mutant 3′-UTR of VCAM1 and VEGFA or cZBTB44 containing the putative target site for miR-578 was inserted into the downstream of the luciferase gene in the pGL3 vectors (Promega, Madison, WI, USA). RF/6A cells were seeded in 24-well plates at the concentration of 2 × 10^5^ cells/well. Two hundred nanograms of pGL3-vector containing corresponding gene sequence were transfected in combination with miR-578 mimic. The luciferase activity assay was conducted 24 h after transfection using the Dual Luciferase Reporter Assay System (Promega). Relative luciferase activity was normalized to *Renilla luciferase* activity internal control.

### Quantitative real-time PCR

Total RNA was extracted from cells, tissues and clinical samples using Trizol reagent (Life Technologies, Carlsbad, CA, USA). To quantify the amount of target mRNA, miRNA and circRNA, cDNAs were synthesized with the PrimeScript RT Master Mix (Takara, Dalian, China). Quantitative analysis of gene expression was conducted using an Applied Biosystems (Grand Island, NY, USA) 7500 Sequence Detection System with the SYBR Premix Ex Taq Ⅱ (Takara, Dalian, China), and gene expression was calculated relative to the internal control GAPDH through the ^ΔΔ^Ct method. The relative target gene levels were presented as the ratio of change versus internal control. The specific primers for the detected genes were listed in Table S1.

### Statistical analysis

All data were expressed as means ± SEM. For normally distributed data, statistical analysis was performed using 2-tailed Student's t test or one-way analysis of variance (ANOVA). For data with non-normal distribution, statistical analysis was performed using the Kruskal-Wallis test. *P < 0.05 was considered statistically significant.

## Results

### cZBTB44 expression is up-regulated in laser-induced CNV lesions and in endothelial cells upon hypoxia stress

**Figure 1:**
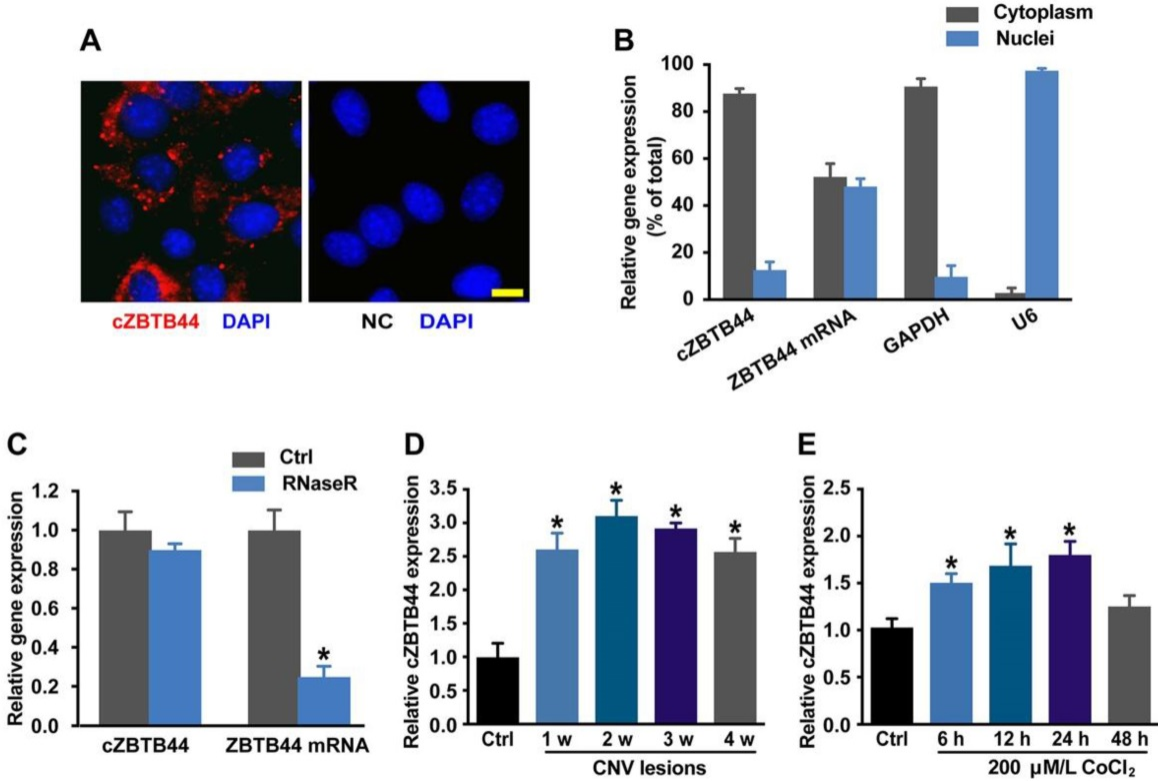
** cZBTB44 expression pattern in CNV lesions and in RF/6Acells upon hypoxia stress.** (A) RNA-FISH assays were conducted to detect cZBTB44 expression distribution in RF/6A cells using Cy3-labeled sense (negative control, NC) and antisense probes (cZBTB44). Nuclei were stained with 4, 6-diamidino-2-phenylindole (DAPI). Scale bar, 10 μm. (B) The expression of nuclear control transcript (U6), cytoplasm control transcript (GAPDH), ZBTB44 mRNA, and cZBTB44 was detected by qRT-PCRs in the nuclei and cytoplasm of RF/6A cells. (C) Total RNAs of RF/6A cells were digested with RNase R. qRT-PCRs were conducted to detect cZBTB44 expression. ZBTB44 mRNA was detected as the RNase R-sensitive control (n=4, *P<0.05 versus corresponding control group). (D) qRT-PCRs were conducted to detect the expression of cZBTB44 in the choroidal samples of C57BL/6 mice after 1, 2, 3, and 4-week laser irradiation (n=5, *P<0.05). (E) RF/6A cells were treatment with 200 μM CoCl_2_ for the indicated time points. qRT-PCRs were conducted to detect cZBTB44 expression (n=5, *P<0.05). All data were from at least three independent experiments.

We first determined whether cZBTB44 was expressed in choroid-retinal endothelial cells (RF/6A) by fluorescence in situ hybridization (FISH) assay and qRT-PCR. The results showed that cZBTB44 was mainly expressed in the cytoplasm of RF/6A cells (Figure 1A-B). We then estimated cZBTB44 stability by treating the total RNAs from RF/6A cells with RNase R. The results showed cZBTB44 was resistant to RNase R digestion, while linear ZBTB44 mRNA was easily degraded (Figure 1C).

We used laser photocoagulation to build a mouse model of CNV and then determined whether cZBTB44 expression was altered in laser-induced CNV membranes *in vivo*. qRT-PCR assays showed that the expression of cZBTB44 in CNV lesions was significantly higher than that in the control choroidal membranes (Figure 1D). By contrast, ZBTB44 mRNA expression was not altered in the CNV lesions (Figure S1). Hypoxia is recognized as a critical driver of CNV formation. RF/6A cells were exposed to the culture medium containing CoCl_2_ to mimic hypoxic condition. We observed that CoCl_2_ treatment led to increased cZBTB44 expression in a time-dependent manner (Figure 1E). Meanwhile, CoCl_2_ treatment had no effect on the expression of ZBTB44 mRNA (Figure S2).

### cZBTB44 regulates CNV development *in vivo*

**Figure 2:**
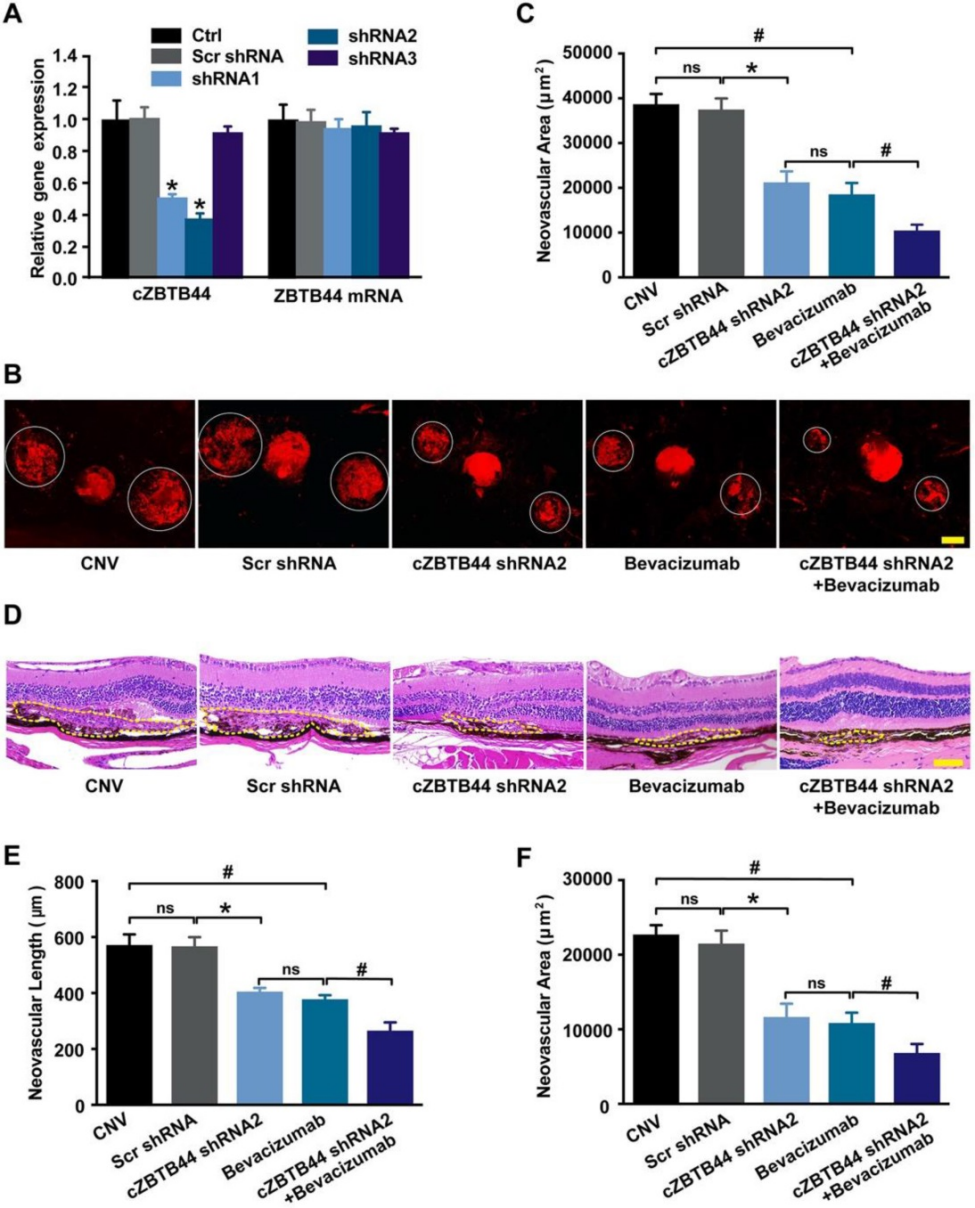
** cZBTB44 silencing suppresses the development of laser-induced CNV *in vivo*.** (A) Eight weeks old C57BL/6 mice received an intravitreal injection of scrambled (Scr) shRNA, cZBTB44 shRNA, or left untreated (Ctrl). qRT-PCRs were conducted to detect cZBTB44 and ZBTB44 mRNA expression in the choroid at day 14 (n=5, *P<0.05 versus Scr shRNA). (B and C) After laser treatment, 6-8 weeks old C57BL/6 mice received an intravitreal injection of scrambled (Scr) shRNA, cZBTB44 shRNA2, bevacizumab, combinations of cZBTB44 shRNA2 and bevacizumab, or left untreated (CNV). On the 14th day, CNV in flat-mounted choroidal tissues was visualized by fluorescent labeling of IB4 and quantification of CNV fluorescence was conducted. White circles denote the lesion areas. Scale bar, 100 μm (n=5, *P<0.05 versus Scr shRNA, **^#^***P*<0.05 versus Bevacizumab, ns: no significance). (D) Representative images of hematoxylin and eosin staining of transverse sections of the CNV 14 days after photocoagulation were shown and quantification of the length (E) and the area (F) of the CNV was performed. Yellow dotted lines denote the lesion areas. Scale bar, 100 μm (n=5, *P<0.05 versus Scr shRNA, **^#^***P*<0.05 versus Bevacizumab, ns: no significance). All data were from at least three independent experiments.

We designed three different short hairpin RNA (shRNAs) to silence cZBTB44 expression. Two shRNAs could significantly reduce cZBTB44 expression in the choroid of mice (Figure 2A). We selected shRNA2 due to its higher silencing efficiency. To investigate the role of cZBTB44 in laser-induced CNV *in vivo*, intravitreal injection of cZBTB44 shRNA2 was administered. Isolectin B4 (IB4) immunofluorescence was employed to label the neovascular area in the choroidal flat-mounts. The results showed that cZBTB44 silencing led to reduced CNV lesion area in laser-induced CNV mouse model, showing a similar effect of anti-VEGF agents including bevacizumab or aflibercept on CNV inhibition. Moreover, cZBTB44 silencing combined with anti-VEGF agents inhibited CNV formation more effectively than their individual effects (Figure 2B-C and Figure S3A-B). Similarly, HE staining analysis showed that cZBTB44 silencing led to decreased CNV lesion length and area (Figure 2D-F). By contrast, cZBTB44 overexpression accelerated CNV development compared with mice injected of vector (Figure S3C-E). Collectively, these results indicate that cZBTB44 is involved in the formation of CNV *in vivo*.

### cZBTB44 regulates CNV development *ex vivo*

**Figure 3:**
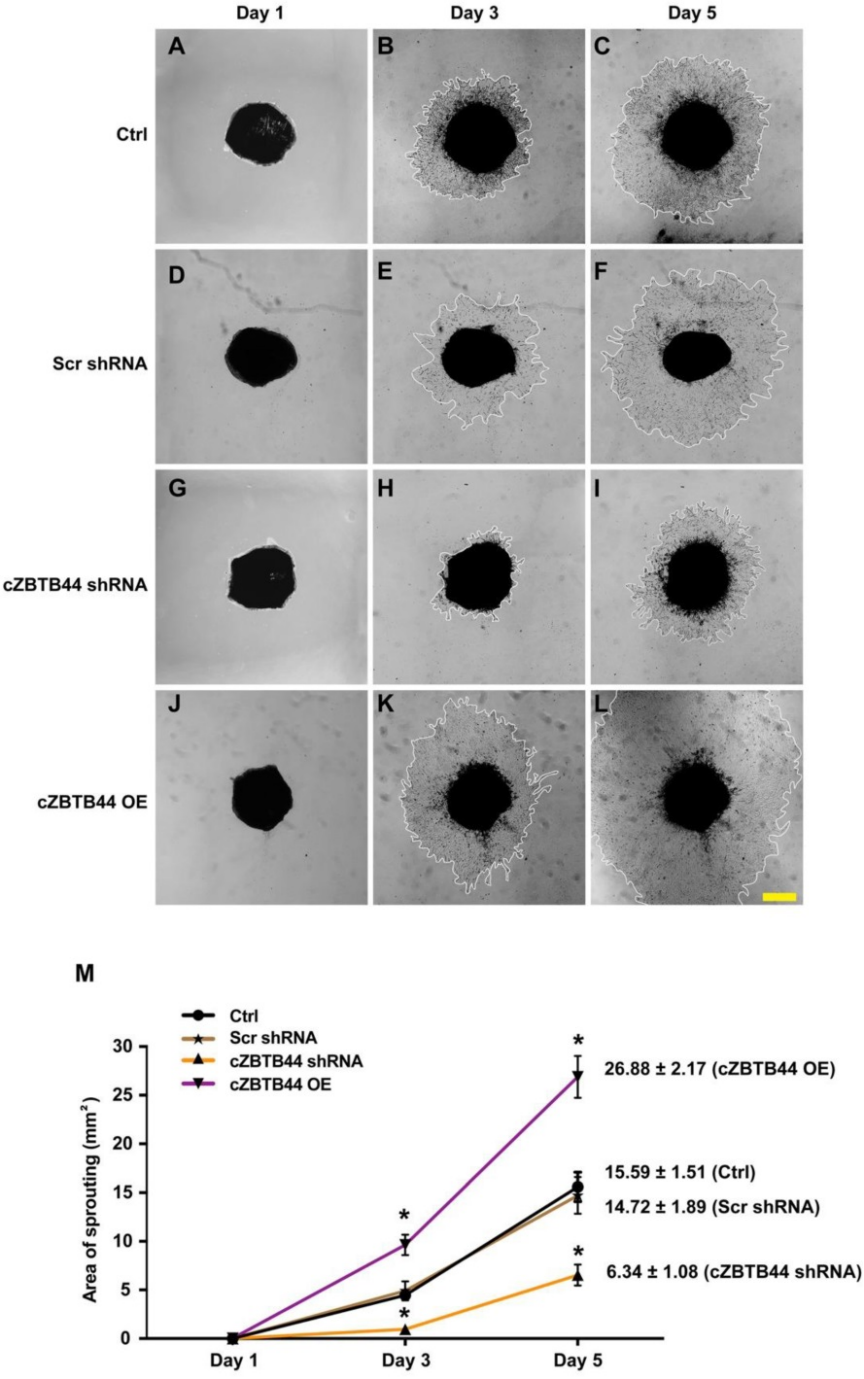
** cZBTB44 regulates CNV development *ex vivo*.** Eight-week-old C57BL/6 mice received an intravitreal injection of scrambled (Scr) shRNA, cZBTB44 shRNA, cZBTB44 overexpression (OE) or left untreated (Ctrl). At day 14, the mouse choroidal sprouting assay was conducted to measure the angiogenic potency of choroidal explants in each group of mice. (A-L) Representative images of the choroidal sprouting areas at indicated time points were shown. Scale bar, 500 μm. (M) Quantification of the areas of the CNV sprouts was shown (n=5-8, *P<0.05 versus Scr shRNA group). All data were from at least three independent experiments.

We further investigated the role of cZBTB44 in choroidal angiogenic activity using an *ex vivo* model 20. The experiments were divided into four groups, cZBTB44 silencing group, cZBTB44 overexpression group, scrambled shRNA group and untreated group. At day 1, day 3 and day 5, the choroidal capillary sprouting area of these explants were photographed. cZBTB44 silencing led to decreased sprouting area (Figure 3A-I). By contrast, cZBTB44 overexpression led to a lager vessel-sprouting area (Figure 3J-L). Quantitative result of the choroidal sprouting areas was shown in Figure 3M. Collectively, the above-mentioned results suggest that cZBTB44 is involved in the regulation of CNV in the *ex vivo* model.

### cZBTB44 regulates endothelial cell function *in vitro*

**Figure 4:**
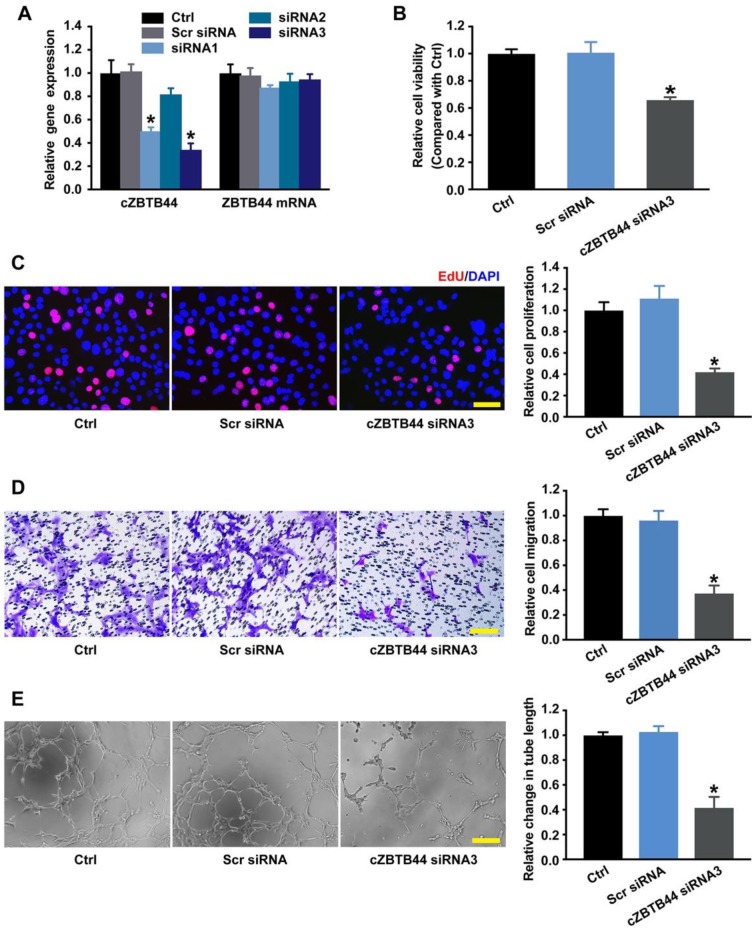
** cZBTB44 regulates endothelial function *in vitro*.** (A) RF/6A cells were transfected with scrambled (Scr) siRNA, siRNA targeting the sequence of cZBTB44, or left untreated (Ctrl) for 36 h. qRT-PCRs were conducted to detect cZBTB44 and ZBTB44 mRNA expression (n=4, *P<0.05 versus Scr siRNA). (B) Cell viability was detected using CCK-8 method (n=5, *P<0.05 versus Scr siRNA). (C) Cell proliferation was detected using EdU detection kit (Ribobio, Guangzhou, China) to analyze the incorporation of EdU in DNA synthesis. Scale bar, 50 μm (n=4, *P<0.05 versus Scr siRNA). (D) Migration of RF/6A cells was measured using transwell assay and the cells that migrated through the transwell were quantified. Scale bar, 100 μm (n=4, *P<0.05 versus Scr siRNA). (E) RF/6A cells were seeded on the matrigel matrix. The tube-like structures were observed 4 h after cell seeding. Average length of tube formation for each field was statistically analyzed. Scale bar, 100 μm (n=4, *P<0.05 versus Scr siRNA). All data were from at least three independent experiments.

Endothelial cells are recognized as major players of ocular neovascular disorders 21, 22. We thus investigated the role of cZBTB44 in endothelial cells *in vitro*. We designed three different siRNAs for cZBTB44 silencing. Both siRNA1 and siRNA3 transfection could significantly reduce cZBTB44 expression (Figure 4A).

We detected the effect of cZBTB44 silencing on RF/6A cell function under both basal condition and hypoxia stress. cZBTB44 silencing by siRNA3 resulted in decreased cell viability (Figure 4B), reduced proliferation (Figure 4C), decelerated cell migration and tube formation (Figure 4D-E). The similar results of cZBTB44 silencing on RF/6A cell function by siRNA1 was showed in Figure S4A-D. Hypoxic stress is tightly associated with CNV development 23, 24. We also revealed that cZBTB44 silencing by siRNA1 or siRNA3 significantly inhibited the viability, proliferation, migration and tube formation of RF/6A cells under hypoxia condition (Figure S5A-D).

We then performed the gain-of-function analysis of cZBTB44, and investigated whether cZBTB44 is sufficient to drive the endothelial phenotype alone. We observed that cZBTB44 overexpression obviously increased the viability and proliferation, and promoted the migration and tube formation of RF/6A cells under both basal condition and hypoxic condition (Figure S6A-D and S7A-D). Taken together, these results indicate cZBTB44 is a crucial regulator of endothelial cell function.

### cZBTB44 regulates endothelial cell function by serving as a miRNA sponge

**Figure 5:**
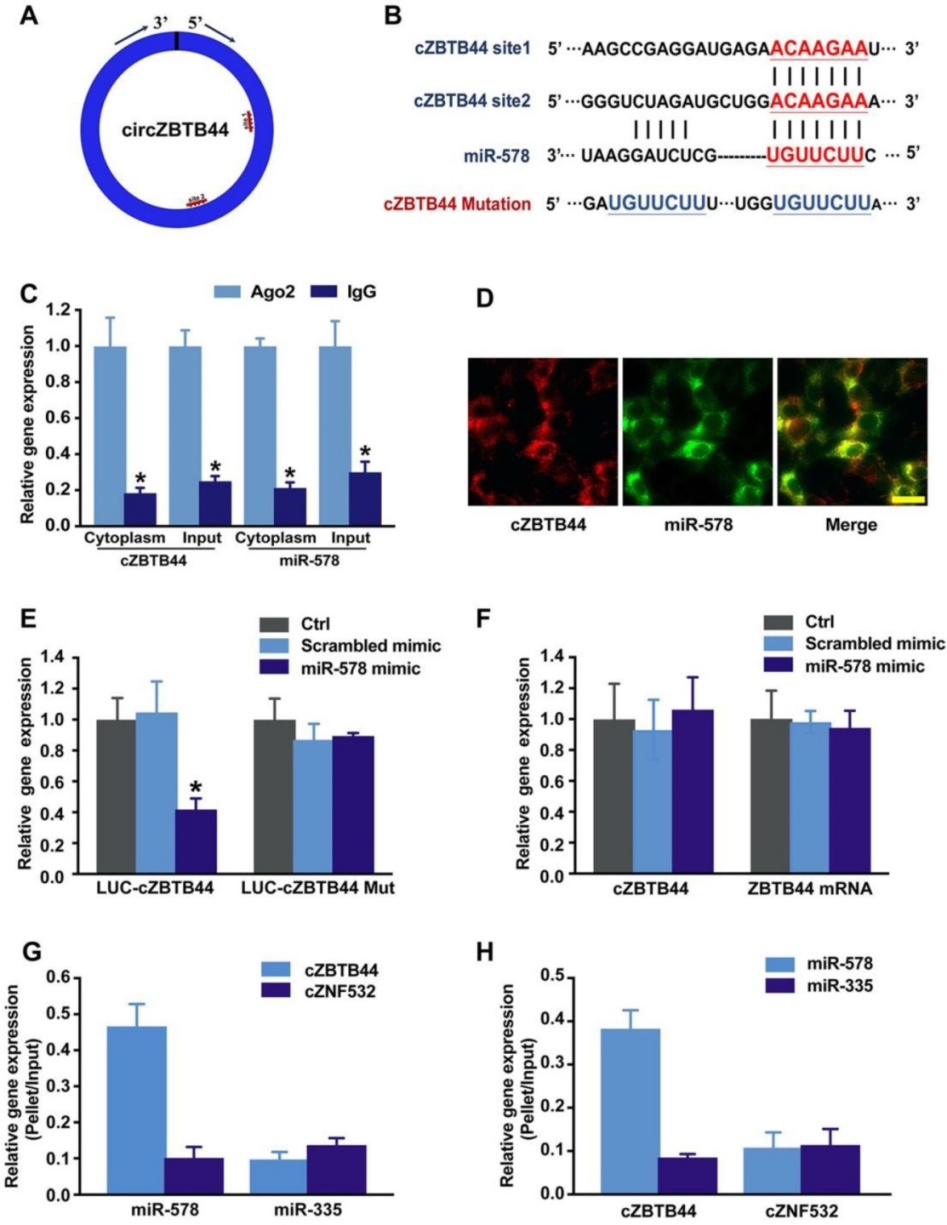
** cZBTB44 serves as a miRNA sponge in regulating endothelial function.** (A) A schematic illustration showing the putative binding sites of miR-578 on cZBTB44. (B) The potential binding sites sequence of miR-578 on cZBTB44. Bottom: mutation in the cZBTB44 sequence to generate the mutant luciferase reporter construct. (C) The cytoplasm and total cellular fractions were isolated from RF/6A cells, and immunoprecipitated using Ago2 or IgG antibody. cZBTB44 and miR-578 amount in the immunoprecipitate was determined by qRT-PCRs (n=4, *P<0.05). (D) RNA-FISH assays were conducted to detect cZBTB44 and miR-578 expression in RF/6A cells. cZBTB44, red; miR-578, green. Scale bar, 20 μm. (E) RF/6A cells were co-transfected LUC-cZBTB44 or LUC-cZBTB44-mutant with miR-578 mimic or scrambled mimic. Luciferase activity was detected 36 h after transfection (n=4, *P<0.05 versus corresponding scrambled mimic). (F) RF/6A cells were transfected with miR-578 mimic, scrambled mimic, or left untreated (Ctrl). qRT-PCRs were conducted to detect cZBTB44 and ZBTB44 mRNA expression. The data was shown as relative change compared with the control group (n=4). (G) The 3'-end biotinylated miR-578 or miR-335 (negative control) duplexes were transfected into RF/6A cells. After streptavidin capture, cZBTB44 and cZNF532 (negative control) expression levels in the input and bound fractions were detected by qRT-PCRs (n=4). (H) The 3'-end biotinylated cZBTB44 or cZNF532 (negative control) were transfected into RF/6A cells. After streptavidin capture, miR-578 and miR-335 (negative control) expression levels in the input and bound fractions were detected by qRT-PCRs (n=4). All data were from at least three independent experiments.

cZBTB44 was mainly expressed in the cytoplasm of RF/6A cells. We speculated that cZBTB44 might regulate gene expression by serving as a miRNA sponge. Circular RNA Interactome database and sequence analysis indicated that there were two potential binding sites for miR-578 in cZBTB44 (Figure 5A-B). The functional chain of the mature miRNA is assembled by Ago2 into the RNA-mediated silencing complex RISC, which directs RISC to silence the target mRNA. We employed RNA immunoprecipitation (RIP) assays to show that cZBTB44 was enriched in Ago2-containing immunoprecipitates in comparison with the control, immunoglobulin G (IgG) immunoprecipitates (Figure 5C). miR-578 expression level in Ago2-containing immunoprecipitates was significantly higher than that in IgG immunoprecipitates (Figure 5C). These results also suggested that both cZBTB44 and miR-578 were mainly localized in the cytoplasm of RF/6A cells. RNA-FISH also confirmed the co-localization between cZBTB44 and miR-578 (Figure 5D). We next inserted cZBTB44 sequence into the downstream of luciferase reporter (LUC-cZBTB44). miR-578 mimic transfection decreased the luciferase activity of LUC-cZBTB44, but had no effect on the luciferase activity of LUC-cZBTB44 mutant (Figure 5E). miR-578 mimic transfection did not affect the expression level of cZBTB44 and ZBTB44 mRNA (Figure 5F), indicating that miR-578 had no effect on the degradation of cZBTB44 and ZBTB44 mRNA. Using biotin-coupled miR-578, we observed higher enrichment of cZBTB44 in miR-578-captured fraction compared to the negative control, biotinylated miR-335 (Figure 5G). We also observed higher enrichment of miR-578 in cZBTB44-captured fraction compared to the negative control, biotinylated cZNF532 (Figure 5H). The above-mentioned results suggest that cZBTB44 serves as a sponge of miR-578 in endothelial cells.

### cZBTB44/miR-578 interaction is involved in regulating endothelial cell function

**Figure 6:**
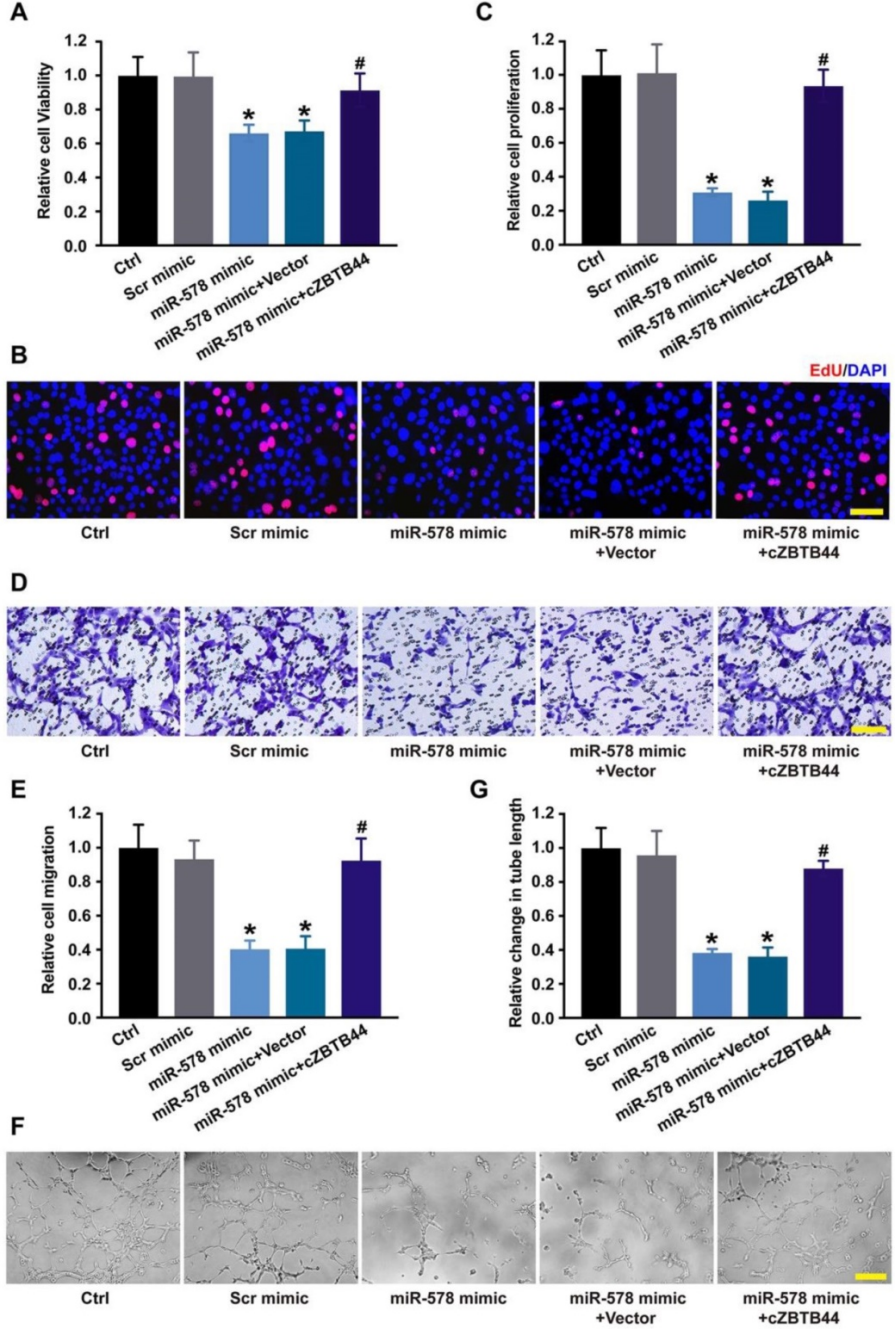
** cZBTB44/miR-578 interaction is involved in regulating endothelial cell function.** (A) RF/6A cells were transfected with scrambled mimic (Scr), miR-578 mimic, miR-578 mimic plus pcDNA3.1-cZBTB44, or pcDNA3.1 (Vector). Cell viability was detected using CCK-8 method (n=5, *P<0.05 versus Scr mimic, **^#^***P*<0.05 versus miR-578 mimic + Vector). (B and C) Cell proliferation was detected using EdU detection kit (Ribobio). Scale bar, 50 μm (n=4, *P<0.05 versus Scr mimic, **^#^***P*<0.05 versus miR-578 mimic + Vector). (D and E) Migration of RF/6A cells was measured using transwell assay and the cells that migrated through the transwell were quantified. Scale bar, 100 μm (n=4, *P<0.05 versus Scr mimic, **^#^***P*<0.05 versus miR-578 mimic + Vector). (F and G) The tube-like structures were observed 4 h after cell seeding on the matrigel matrix. Average length of tube formation for each field was statistically analyzed. Scale bar, 100 μm (n=4, *P<0.05 versus Scr mimic, **^#^***P*<0.05 versus miR-578 mimic + Vector). All data were from at least three independent experiments.

Since miR-578 was sponged by cZBTB44, we then investigated the role of miR-578 in regulating the function of RF/6A cells. miR-578 mimic transfection decreased the proliferation, migration and tube formation ability of RF/6A cells, while cZBTB44 overexpression could rescue the effects of miR-578 mimic transfection on RF/6A cell function (Figure 6A-G). We further revealed that cZBTB44 siRNA plus miR-578 mimic transfection inhibited endothelial cell viability, proliferation, migration and tube formation (Figure S8). miR-578 inhibitor transfection significantly promoted endothelial cell proliferation, migration and tube formation (Figure S9). The results suggest that cZBTB44/miR-578 interaction participates in the regulation of endothelial cell function.

### cZBTB44/miR-578 interaction is involved in regulating CNV development

**Figure 7:**
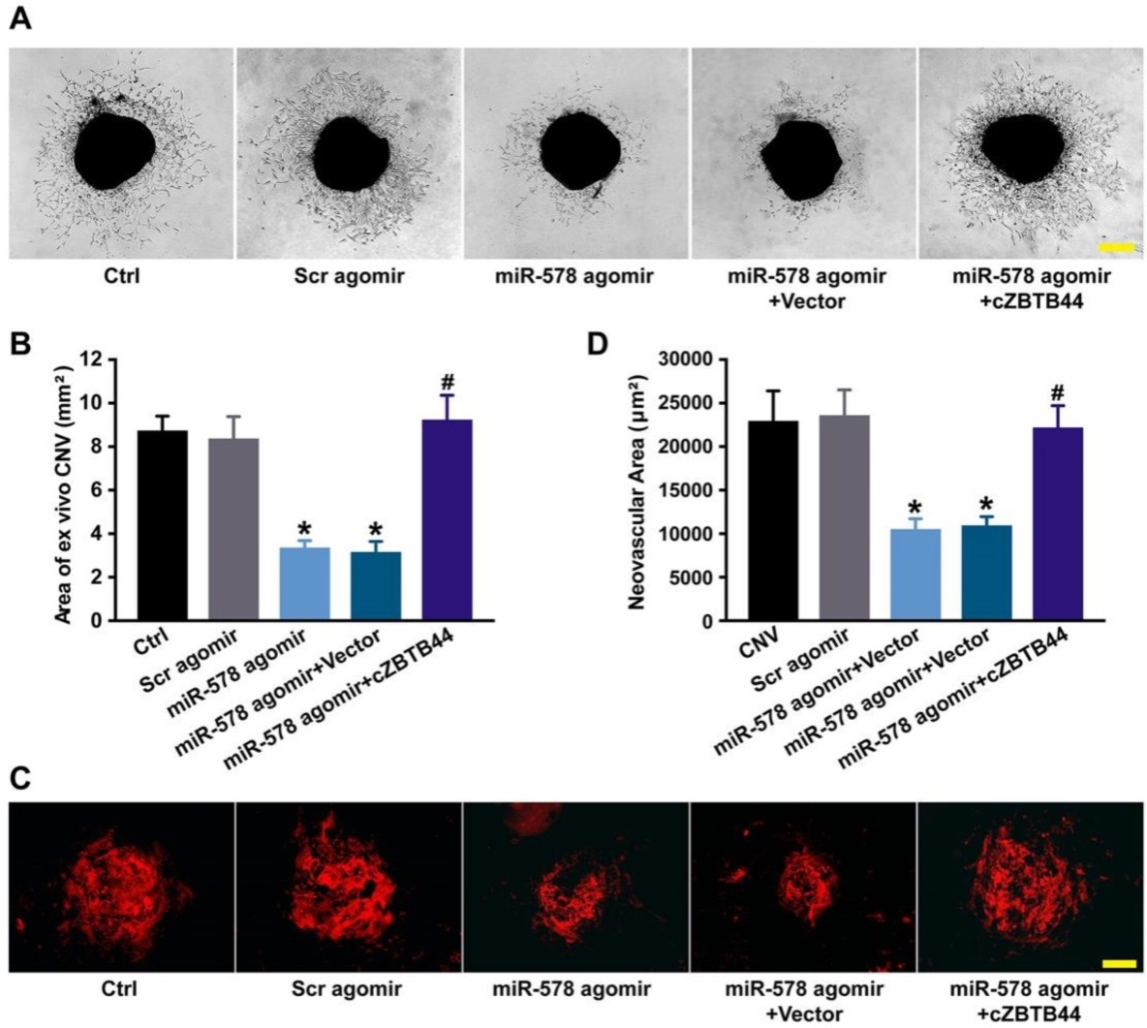
** cZBTB44/miR-578 interaction is involved in regulating CNV development.** (A and B) Eight-week-old C57BL/6 mice received an intravitreal injection of Scr agomir, miR-578 agomir, miR-578 agomir plus AAV-cZBTB44, or left untreated (Ctrl). The mouse choroidal sprouting assay was conducted to measure the angiogenic potential of choroidal explants in each group of mice. Representative images of the choroidal sprouting areas were performed. Quantification of the areas of the CNV sprouts was shown. Scale bar, 500 μm. (n=5, *P<0.05 versus Scr agomir, **^#^***P*<0.05 versus miR-578 agomir + Vector). (C and D) After laser treatment, mice were treated as shown. At day 14, CNV in flat-mounted choroidal tissues was visualized by IB4 staining and quantification of CNV fluorescence was conducted. Scale bar, 100 μm (n=5, *P<0.05 versus Scr agomir, **^#^***P*<0.05 versus miR-578 agomir + Vector). All data were from at least three independent experiments.

We also investigated the role of miR-578 in CNV development. miR-578 up-regulation by agomir injection could mimic the effect of cZBTB44 silencing on CNV formation, showing pronounced anti-angiogenic activity both *ex vivo* and *in vivo* (Figure 7A-D). We further explored whether the addition of exogenous cZBTB44 could overwhelm the inhibitory effect of miR-578. The results showed that exogenous cZBTB44 could block the regulation of miR-578 in CNV (Figure 7A-D). Therefore, we conclude that cZBTB44/miR-578 interaction is involved in regulating CNV development.

### cZBTB44-miR-578-VEGFA/VCAM1 network regulates endothelial angiogenic effect

**Figure 8:**
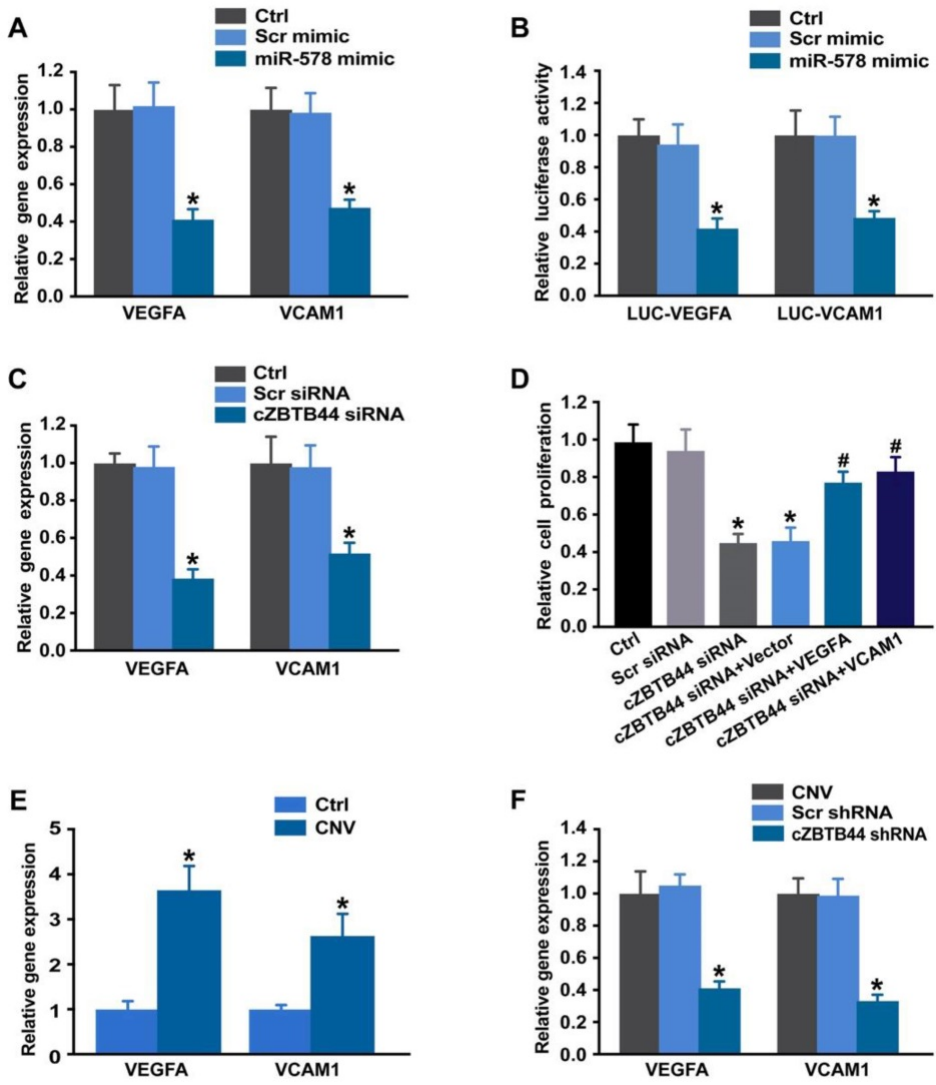
** cZBTB44-miR-578-VEGFA/VCAM1 network is involved in regulating CNV development.** (A) RF/6A cells were transfected with Scr mimic, miR-578 mimic, or left untreated (Ctrl). qRT-PCRs were conducted to detect VEGFA and VCAM1 expression (n=4, *P<0.05 versus Scr mimic). (B) RF/6A cells were co-transfected LUC-VEGFA and LUC-VCAM1 with miRNA mimics. Luciferase activity was detected 36 h after transfection (n=4, *P<0.05 versus Scr mimic). (C) RF/6A cells were transfected with Scr siRNA, cZBTB44 siRNA, or left untreated (Ctrl) for 36 h. qRT-PCRs were conducted to detect VEGFA and VCAM1 expression (n=4, *P<0.05 versus Scr siRNA). (D) RF/6A cells were treated as shown. Cell proliferation was determined using EdU detection kit (Ribobio) (n=4, *P<0.05 versus Scr siRNA, **^#^***P*<0.05 versus cZBTB44 siRNA + Vector). (E) qRT-PCRs were conducted to detect VEGFA and VCAM1 expression in the choroid of untreated mice (Ctrl) and CNV mice (n=5, *P<0.05 versus Ctrl group). (F) Eight-week-C57BL/6 mice received an intravitreal injection of Scr shRNA, cZBTB44 shRNA, or left untreated (Ctrl) 3 after laser treatment. qRT-PCRs were conducted to detect VEGFA and VCAM1 expression in the choroid at day 14 after laser treatment (n=5, *P<0.05 versus Scr shRNA). All data were from at least three independent experiments.

Targetscan database was used to predict the target genes of miR-578. The candidate genes including VEGFA and VCAM1 were identified, which have been reported to be involved in angiogenesis 25, 26. miR-578 mimic transfection significantly inhibited VEGFA and VCAM1 expression in RF/6A cells (Figure 8A). By contrast, the expression of other angiogenic factors, fibroblast growth factor 2 (FGF2), platelet-derived growth factor (PDGF), NOTCH1, C-X-C motif chemokine receptor 4 (CXCR4), and matrix metalloproteinase 9 (MMP9) was not affected by miR-578 mimic transfection (Figure S10). Subsequently, luciferase reporter assay was used to verify direct regulation of miR-578 on its angiogenic targets. miR-578 mimic transfection decreased the luciferase activities of reporter constructs containing target sequences of VEGFA and VCAM1 (Figure 8B). These results suggest that miR-578 is a direct regulator of VEGFA and VCAM1 in RF/6A cells.

We also showed that cZBTB44 silencing significantly reduced VEGFA and VCAM1 expression (Figure 8C). Further functional analysis revealed that VEGFA and VCAM1 overexpression could partially rescue the repressive effect of cZBTB44 silencing on RF/6A cell proliferation, migration, and tube formation (Figure 8D and Figure S11). In laser-induced CNV, the expression of VEGFA and VCAM1 was significantly up-regulated (Figure 8E). cZBTB44 silencing significantly decreased VEGFA and VCAM1 expression (Figure 8F). We thus conclude that cZBTB44-miR-578-VEGFA/VCAM1 crosstalk is involved in regulating endothelial cell function.

### Clinical relevance of cZBTB44 in the patients with CNV

**Figure 9:**
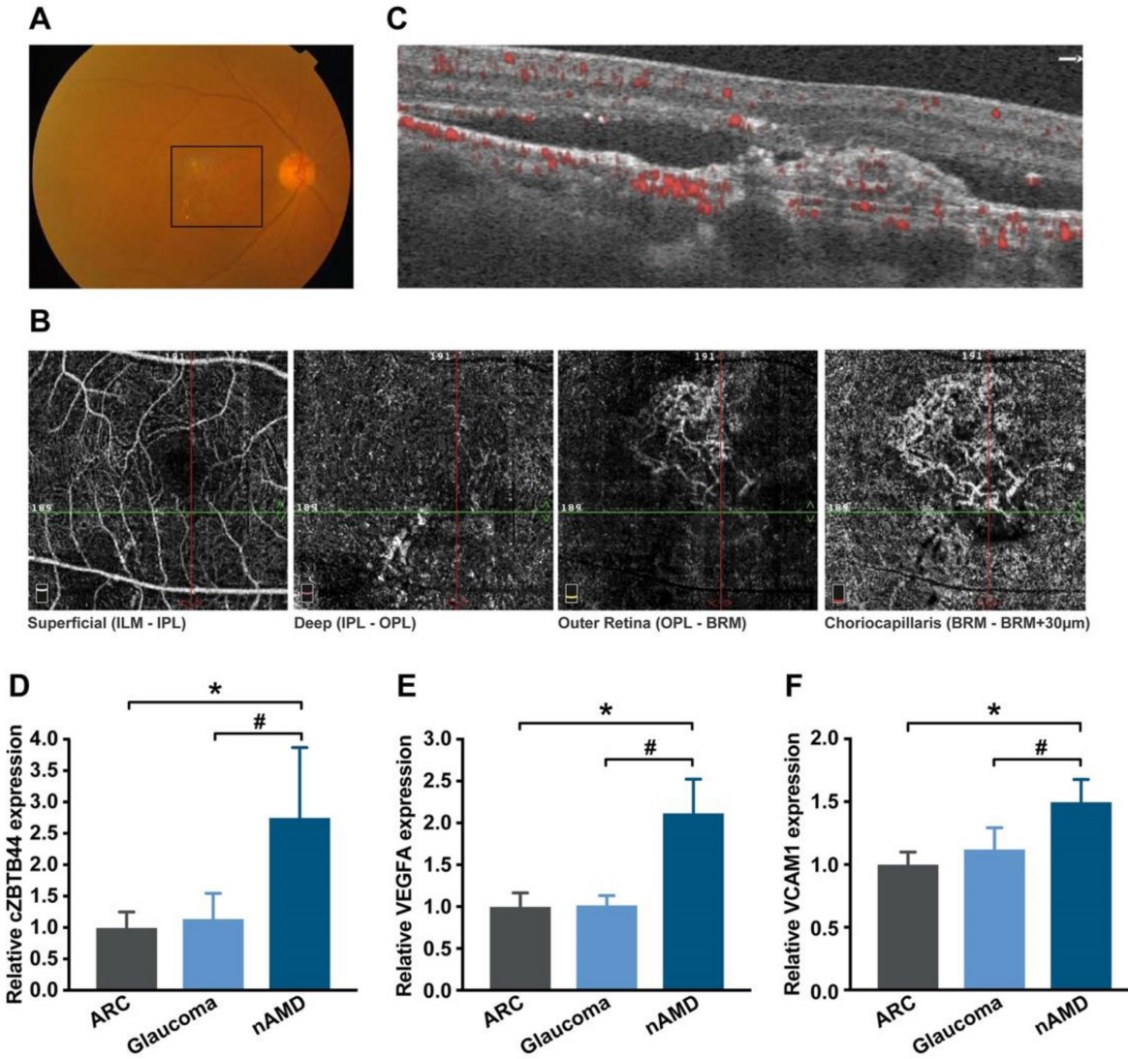
** Clinical relevance of cZBTB44 in CNV patients.** (A) Ocular fundus photography was used to show the clinical characters of CNV patients. The black square outlined the lesion area. (B and C) The CNV network was observed by optical coherence tomography angiography (OCTA). Structural OCT (C) indicated that CNV was predominantly in the outer retina and choriocapillaris, with corresponding en face structural OCTA images (B, image size 3 × 3 mm). The dashed green line showed the location of the cross section. (D-F) qRT-PCRs were conducted to detect the expression of cZBTB44, VEGFA and VCAM1 in the AH of patients with nAMD, ARC, and glaucoma (n=15, *P<0.05 versus ARC, **^#^***P*<0.05 versus Glaucoma). All data were from at least three independent experiments.

The neovascular AMD is characterized by the presence of CNV. To translate our findings to a physiologically relevant context, we performed qRT-PCR to detect the expression level of cZBTB44 in the aqueous humor (AH) and plasma fraction of the patients with nAMD. AH, an important body fluid in the eye, is known to be correlated with plenty of ocular diseases 27. Fundus photograph showed that there was gray-yellow newly formed choroidal vessels in nAMD patients, along with subretinal hemorrhage and intraretinal fluid (Figure 9A). Optical coherence tomography angiography (OCTA) showed an abnormal neovascular network in the macula that was predominantly in the outer retina and choriocapillaris (Figure 9B-C). The expression of cZBTB44, VEGFA and VCAM1 was significantly up-regulated in the AH of nAMD patients, while there was no increase in the AH of patients with age-related cataract (ARC) and glaucoma (Figure 9D-F and Table S2). There was no significant difference in plasma cZBTB44 expression in nAMD patients compared with ARC and glaucoma patients (Figure S12). Collectively, these results suggest that cZBTB44 is potentially involved in the pathogenesis of neovascular AMD.

## Discussion

Although circRNAs have been discovered for decades, they are thought to be errors in RNA splicing 28. With the advancements in high throughput sequencing technologies and bioinformatics, circRNAs are now recognized as ubiquitously expressed RNA molecules with a variety of biological activities, including sponge, translation, biomarker, and regulation molecules 29, 30. Existing studies have highlighted the importance of circRNA dysregulation in a multitude of human diseases 31. In this study, we show that cZBTB44 expression is significantly up-regulated in laser-induced CNV and in endothelial cell upon hypoxia stress. cZBTB44 silencing decreases CNV development *in vivo*. cZBTB44 also regulates endothelial cell function both *in vitro* and in an *ex vixo* model of choroidal angiogenesis. Mechanistically, cZBTB44 works as an endogenous sponge by binding to miR-578 and consequently represses miR-578 activity, resulting in increased VEGFA and VCAM1 expression. This study provides a novel insight for understanding the pathogenesis of CNV.

CNV is the main characterization of exudative AMD, which is the leading cause of vision loss in industrialized countries 3, 32. The establishment of experiment animal models is an important prerequisite for studying the cellular and molecular mechanisms involved in the pathogenesis of CNV 33. The occurrence of laser-induced CNV are based on rupturing of RPE-Bruch' membrane complex, which leads to the generation of new blood vessels from the choroid into the sub-retinal space or penetrating through Bruch's membrane. Because the mouse model of laser-induced CNV and human exudative AMD share the similar characteristics, this model has been extensively used to explore the molecular mechanism or new potential treatment targets of CNV 33. This study also employed this model to study the effect of cZBTB44 molecular on CNV development. cZBTB44 expression is significantly up-regulated in laser-induced CNV and cZBTB44 silencing can retard the development of CNV-induced by laser. In contrast, cZBTB44 overexpression remarkably promotes the development of CNV. In addition, cZBTB44 expression in the clinical samples of CNV patients is much higher than that in patients with cataract or glaucoma. Thus, we speculate that cZBTB44 is involved in the regulation of CNV *in vivo*.

CNV is a class of the pathologic angiogenesis in the eye. The formation of angiogenesis involves vascular endothelial cell activation, extracellular matrix degradation, endothelial cell migration and proliferation, formation of tight junctions, recruitment of pericytes, and deposition of new basement membrane. Among them, endothelial cells play a leading role during the angiogenic process 34. Activated endothelial cells produce multiple angiogenesis-related factors, proliferate and migrate through Bruch' membrane and RPE layers, ultimately causing the formation of CNV 22. Thus, we performed CCK-8 assay, EdU staining, cell migration assay and tube formation assay to examine the effects of cZBTB44 on endothelial cell function, further to elucidate whether cZBTB44 regulates the occurrence and development of CNV. *In vitro* studies reveal that increased cZBTB44 is tightly associated with abnormal endothelial cell proliferation, migration and tube formation. These activities are coincident with the properties of endothelial cells involving in neovascularization.

Choroidal capillary sprouting assay, an *ex vivo* mouse model of microvascular angiogenesis, is highly reproducible 20. This sprouting model using choroidal tissue can be used to evaluate angiogenic potential of pharmacologic compounds or certain genes for microvascular disease research. We also performed the model to further confirm that cZBTB44 regulates the function of microvascular endothelial cells during the formation of CNV. Thus, it is not surprising that cZBTB44 is involved in the pathogenesis of microvascular angiogenesis.

We further investigated the mechanisms of cZBTB44-mediated angiogenic function. Generally, exonic circRNAs are predominantly localized in cytoplasm, which contain miRNA response elements (MREs) and can serve as competitive endogenous sponge RNAs to compete for miRNA-binding sites. As a result, the expression level of miRNA target genes would change 11, 35. It has been clearly demonstrated that ciRS-7/CDR1as and Sry circRNA act as inhibitors of miRNA activity via binding with miR-7 and mi-138, respectively 36. cZNF609 has been reported to function as an endogenous miR-615-5p sponge to sequester and inhibit miR-615-5p activity 37. However, whether acting as a ceRNA sponge is a general function of circular RNAs remains controversial. Giuseppe Militello G selected six circular RNAs based on their high numbers of AGO-bound regions in the circBase database to prove that these circRNAs may not function as miRNA sponges 38. Another work identified that the sequence of orthologous circRNAs is no higher than their neighboring linear exons and recognized that a large majority of circRNAs are inconsequential side-products of pre-mRNA splicing 39. A recent bioinformatics study found that circRNA exons tend to be depleted of polymorphisms at predicted miRNA binding sites, suggesting that some circRNAs indeed function as miRNA sponges 40. In addition, thousands of conserved miRNAs binding sites overlap with the circularizing portions of transcripts that generate from circRNAs in Drosophila, which support the notion of circRNAs as miRNAs decoys 41. Herein, we determined the relative expression abundance of cZBTB44 and miR-578 in the cytoplasm of RF/6A cells. The results show that cZBTB44 has a similar expression abundance as miR-578. Increased cZBTB44 may sponge and sequester miR-578, ameliorating miR-578-mediated repressive effects. cZBTB44/miR-578/targets constitute a regulatory network. A slight change in cZBTB44 level may alter miR-578-mediated network. This network provides more precise gene regulation during the process of ocular neovascularization.

VEGFA and VCAM1 were identified as the target genes of miR-578. The role of VEGFA has been confirmed in many ocular diseases, such as nAMD, diabetic retinopathy, as well as neovascular glaucoma 42. VCAM1, an Ig-like intercellular adhesion molecule, increases the susceptibility of patients to oxidative stress. Oxidative stress promotes the pro-angiogenic environment in the eye and contributes to retinal neovascularization and CNV 43. It has been shown that miR-578 mimic transfection in HEK293 cells leads to a lower expression of VEGFA. miR-578 is a potential player in BRCA-related breast cancer angiogenesis 44. Our results show that cZBTB44 silencing significantly decreases the expression of VEGFA and VCAM1. VEGFA or VCAM1 overexpression can partially reverse cZBTB44 silencing mediated effects on microvascular endothelial cell function. During pathological angiogenesis, cZBTB44 overexpression becomes the sink of miR-578, and releases the miR-578 mediated inhibitory effect on VEGFA and VCAM1 expression. cZBTB44-miR-578-VEGFA/VCAM1 network is involved in the regulation of CNV development.

The emergence of circular RNAs as regulators of gene expression has undoubtedly altered our understanding of the mechanisms of microvascular neovascularization. This study provides clear evidence for a crucial role of cZBTB44 in mediating CNV development.

## Conclusions

This study reveals a previously undocumented, central role of cZBTB44 involving in CNV development. cZBTB44 silencing suppresses CNV development *in vivo* and *ex vivo*, and inhibits endothelial cell proliferation, migration, tube formation *in vitro*. The regulatory effect of cZBTB44 on CNV development is mediated by acting as an endogenous miR-578 sponge. These results will be valuable for the better understanding of CNV formation and the development of novel cZBTB44 targeted therapies for treating the debilitating ocular diseases caused by neovascularization.

## Abbreviations

AH: aqueous humor
ARC: age-related cataract
circRNA: circular RNA
CNV: choroidal neovascularization
CXCR4: C-X-C motif chemokine receptor 4
IB4: Isolectin B4
MREs: miRNA response element
nAMD: neovascular age-related macular degeneration
OCTA: optical coherence tomography angiography
RPE: retinal pigment epithelium
RIP: RNA immunoprecipitation
siRNA: small interfering RNA
shRNA: small hairpin RNA
VEGF: vascular endothelial growth factor
VCAM1: vascular cell adhesion molecule-1
