# Supplementary material for: Circular RNA-ZBTB44 regulates the development of choroidal neovascularization
Source: Theranostics. 2020 Feb 10;10(7):3293–307. doi: 10.7150/thno.39488 (PMC7053208; doi:10.7150/thno.39488)
Supplement: Supplementary file 1 — Supplementary materials and methods, figures, and tables. [file thnov10p3293s1.pdf]

Supplementary Materials and Methods

Intravitreal administration of anti-VEGF drugs

Intravitreal injections were performed in a standardized manner in anesthetized mice. Anti-VEGF drugs were administered once, immediately after pulse application, and performed under an operating microscope (Zeiss Opmi 6S Microscope; Carl Zeiss Microscopy GmbH, Oberkochen, Germany). After penetration of the sclera approximately 1.5 mm below the limbus in a slightly oblique direction, the beveled needle tip of a Hamilton microsyringe was placed intravitreally in the retrolental space of the eye. During the placement of the needle, some outflow of the vitreous occurs due to the beveled configuration, which greatly reduces the risk of subsequent drug reflux. Then approximately 1.5 μL of either Aflibercept (10 μg/μL, Eylea, Bayer HealthCare) or 1 μL of Bevacizumab (25 μg/μL, Avastin) were injected into the vitreous cavity, followed by 1min holding in place.

Intravitreal AAV administration

C57BL/6J mice (6-8 weeks old, male) were anesthetized by by intraperitoneal injection of a mixture of 40 mg/kg ketamine and 5 mg/kg xylazine, followed by pupil dilation with 0.5% tropicamide and 2.5% phenylephrine (Mydrin-P; Santen, Osaka, Japan). After laser photocoagulation, approximately 1.5 μL (5×10^9 viral genomes/mL) of adeno-associated virus 2 (AAV2) carrying cZBTB44, cZBTB44-shRNA or scrambled-shRNA was delivered into the vitreous using a 33-gauge needle (Hamilton, Bonaduz, Switzerland) while visualizing the fundus directly using an operating

microscope (Zeiss Opmi 6S Microscope; Carl Zeiss Microscopy GmbH, Oberkochen, Germany).

Cell viability assay

Cell viability was determined using Cell Counting Kit-8 (CCK-8) assay. Briefly, RF/6A cells (5×10^3 cells/well) were seeded onto 96-well plates. After required treatment, 10 μL CCK-8 solution (Sigma, St. Louis, MO, USA) was added into each well for an additional 3 h at 37 ℃. The absorbance at a wavelength of 450 nm was measured by a microplate reader (Molecular Device, Sunnyvale, CA, USA).

Cell proliferation assay

EdU (5-ethynyl-2'-deoxyuridine) DNA Cell Proliferation Kit (RiboBio, Guangzhou, China) was used to determine the proliferation of RF/6A cells. After the required treatment, RF/6A cells were incubated with 50 μM EdU for 2 h. They were then fixed with 4% PFA, permeabilized with 0.5% Triton X-100 and incubated with Apollo Dye Solution for labeling the proliferating cells. Cell nuclei were stained using Hoechst (RiboBio, Guangzhou, China).

Cell migration assay

Cell migration ability was tested by 24-well Transwell units. In brief, RF/6A cells were seeded onto the upper well for 16 h, and allowed to invade through the Transwell plate. Migrated cells on the inserts were fixed with 4% PFA, stained with crystal violet, and observed by a microscope. Migration rate was expressed as the fold change in the number of migrated cells through the Transwell plate.

Tube formation assay

The formation of capillary-like structures was detected in a 48-well plate using Matrigel (BD Biosciences, Cat. 356234). 2 × 10^5 RF/6A cells per well were plated onto Matrigel (200 μL). After 4 h culture, the tube length was observed using a light microscope (Nikon, Tokyo, Japan) and quantified using Image J software.

Preparation of nuclear and cytoplasmic fraction

NE-PER Nuclear and Cytoplasmic Extraction Reagents (Thermo Scientific) were utilized to extract the nuclear and cytoplasmic fractions according to the manufacturer's instructions. Briefly, RF/6A cells were cultured in 150-mm dishes for 48 h to achieve about 90% confluence. About 2 × 10^7 cells were harvested and centrifuged at 1,000 rpms for 5 min followed by washing with cold 1 × PBS buffer. The pellet was resuspended and incubated with Cytoplasmic Extraction Reagent I. After cell membrane was disrupted by vigorous vortexing for 15 s, the preparation was incubated on ice for 10 min. Subsequently, the Cytoplasmic Extraction Reagent II was added followed by vortexing for 5 s and incubation for 3 min. The nuclear and cytoplasmic fractions were isolated by centrifuging at 1,500 × g for 5 min.

RNA fluorescent *in situ* hybridization (RNA-FISH)

To detect cZBTB44 expression, RF/6A cells were fixed in 4% PFA, and dehydrated following continuous incubation in 70%, 80%, 95%, and 100% ethanol. Fluorescent probes were heat denatured and incubated with RF/6A cells in a humidified chamber at 37 °C overnight. The cells were then washed with saline sodium citrate buffer. The nuclei were stained with DAPI. Cy3-labeled sense (negative control) and antisense probes were used for RNA-FISH analysis.

Supplementary Figure Legends

Figure S1: Laser irradiation has no effect on ZBTB44 mRNA expression

qRT-PCRs were conducted to detect the expression of ZBTB44 mRNA in the choroidal samples of C57BL/6 mice after 1, 2, 3, and 4-week laser irradiation (n=5). All data were from at least three independent experiments.

Figure S2: $CoCl_2$ treatment has no effect on ZBTB44 mRNA expression

qRT-PCRs were conducted to detect the expression of ZBTB44 mRNA in RF/6A cells, which were incubated with 200 μM $CoCl_2$ for the indicated time points (n=5). All data were from at least three independent experiments.

Figure S3: cZBTB44 regulates CNV development in vivo

(A and B) After laser treatment, 6-8 weeks old C57BL/6 mice received an intravitreal injection of scrambled (Scr) shRNA, cZBTB44 shRNA2, aflibercept, combinations of cZBTB44 shRNA2 and aflibercept. At day 14, CNV in flat-mounted choroidal tissues was visualized by fluorescent labeling of IB4 and quantification of CNV fluorescence was conducted. White circles denoted the lesion areas. Scale bar, 100 μm (n=5, $^{*}P<0.05$ versus Scr shRNA, $^{\#}P<0.05$ versus aflibercept, ns: no significance). (C) Eight weeks old C57BL/6 mice received an intravitreal injection of scrambled-AAV (Vector), cZBTB44 overexpression (OE)-AAV, or left untreated (Ctrl). qRT-PCRs were conducted to detect cZBTB44 expression in the choroid at day 14 (n=4, $^{*}P<0.05$ versus Vector) (D) Eight-week-old C57BL/6 mice received an intravitreal injection of scrambled-AAV (Vector), or cZBTB44 overexpression (OE)-AAV immediately after laser irradiation. At day 14, CNV in flat-mounted choroidal tissues was visualized by fluorescent labeling of IB4 and quantification of CNV fluorescence was conducted.

White circles denoted the lesion areas. Scale bar, 100 μm (n=5, **P*<0.05 versus Vector). (E) Representative images of hematoxylin and eosin staining of paraffin cross sections were shown and quantification of CNV area was performed. Yellow dotted lines denoted the lesion areas. Scale bar, 100 μm (n=5, **P*<0.05 versus Vector). All data were from at least three independent experiments.

Figure S4: cZBTB44 silencing by siRNA1 affects endothelial cell function under basal condition in vitro

RF/6A cells were transfected with Scr siRNA, cZBTB44 siRNA1, or left untreated (Ctrl) for 36 h. (A) Cell viability was detected using CCK-8 method (n=5, **P*<0.05 versus Scr siRNA). (B) Cell proliferation was detected using EdU detection kit (n=4, **P*<0.05 versus Scr siRNA). (C) Migration of RF/6A cells was measured using transwell assay and quantification of migrated cells was performed (n=4, **P*<0.05 versus Scr siRNA). (D) RF/6A cells were seeded on the matrigel matrix. The tube-like structures were observed 4 h after cell seeding. Average length of tube formation for each field was statistically analyzed (n=4, **P*<0.05 versus Scr siRNA). All data were from at least three independent experiments.

Figure S5: cZBTB44 silencing affects endothelial cell function under hypoxic condition in vitro

RF/6A cells were transfected with Scr siRNA, cZBTB44 siRNA1, cZBTB44 siRNA3 or left untreated (Ctrl) and then treated with or without 200 μM $CoCl_2$ to mimic hypoxic stress for 48 h. (A) Cell viability was detected using CCK-8 method (n=5, **P*<0.05 versus Scr siRNA). (B) Cell proliferation was detected using EdU detection kit (n=4,

*$P<0.05$ versus Scr siRNA). (C) Migration of RF/6A cells was measured using transwell assay and quantification of migrated cells was performed (n=4, *$P<0.05$ versus Scr siRNA). (D) The tube-like structures were observed 4 h after cells seeding on the matrix. Average length of the capillary-like tubular structures for each field was statistically analyzed (n=4, *$P<0.05$ versus Scr siRNA). All data were from at least three independent experiments.

Figure S6: cZBTB44 overexpression affects endothelial cell function under basal condition in vitro

RF/6A cells were transfected with pcDNA3.1 (vector), pcDNA3.1-cZBTB44, or left untreated (Ctrl) for 36 h. (A) qRT-PCRs were conducted to detect cZBTB44 expression (n=4, *$P<0.05$ versus Vector group). (B) Cell viability was detected using CCK-8 method (n=5, *$P<0.05$ versus Vector group). (C) Cell proliferation was detected using EdU detection kit (n=4, *$P<0.05$ versus Vector group). (D) Migration of RF/6A cells was measured using transwell assay and quantification of migrated cells was performed (n=4, *$P<0.05$ versus Vector group). (E) The tube-like structures were observed 4 h after cells seeding on the matrix. Average length of the capillary-like tubular structures for each field was statistically analyzed (n=4, *$P<0.05$ versus Vector group). All data were from at least three independent experiments.

Figure S7: cZBTB44 overexpression affects endothelial cell function under hypoxic condition in vitro

RF/6A cells were transfected with pcDNA3.1 (vector), pcDNA3.1-cZBTB44, or left untreated (Ctrl) for 24 h and then treated with or without 200 μM $CoCl_2$ to mimic

hypoxic stress for 48 h. (A) Cell viability was detected using CCK-8 method (n=5, *P<0.05 versus Vector group). (B) Cell proliferation was detected using EdU detection kit to analyze the incorporation of Edu in DNA synthesis (n=4, *P<0.05 versus Vector group). (C) Transwell assay and quantification analysis were conducted to detect the migration of RF/6A cells (n=4, *P<0.05 versus Vector group). (D) The tube-like structures were observed 4 h after cells seeding on the matrix. Average length of the capillary-like tubular structures for each field was statistically analyzed (n=4, *P<0.05 versus Vector group). All data were from at least three independent experiments.

Figure S8: miR-578/cZBTB44 interaction is involved in regulating endothelial cell function

(A) RF/6A cells were co-transfected Scr siRNA, cZBTB44 siRNA, or left untreated (Ctrl) with miRNA mimic for 36 h. Cell viability was detected using CCK-8 method (n=5, *P<0.05 versus Scr siRNA, #P<0.05 versus cZBTB44 siRNA+Scr mimic). (B) Cell proliferation was detected using EdU detection kit (n=4, *P<0.05 versus Scr siRNA, #P<0.05 versus cZBTB44 siRNA+Scr mimic). (C) Migration of RF/6A cells was measured using transwell assay and quantification of migrated cells was performed (n=4, *P<0.05 versus Scr siRNA, #P<0.05 versus cZBTB44 siRNA+Scr mimic). (D) RF/6A cells were seeded on the matrigel matrix. The tube-like structures were observed 4 h after cell seeding. Average length of tune formation for each field was statistically analyzed (n=4, *P<0.05 versus Scr siRNA, #P<0.05 versus cZBTB44 siRNA+Scr mimic). All data were from at least three independent experiments.

Figure S9: miR-578 inhibitor transfection affects endothelial cell function

(A) RF/6A cells were transfected with scrambled inhibitor (Scr), miR-578 inhibitor, or left untreated (Ctrl) for 36 h. Cell viability was detected using CCK-8 method (n=5, **P*<0.05 versus Scr inhibitor). (B) Cell proliferation was detected using EdU detection kit (n=4, **P*<0.05 versus Scr inhibitor). (C) Migration of RF/6A cells was measured using transwell assay and quantification of migrated cells was performed (n=4, **P*<0.05 versus Scr inhibitor). (D) RF/6A cells were seeded on the matrigel matrix. The tube-like structures were observed 4 h after cell seeding. Average length of tube formation for each field was statistically analyzed (n=4, **P*<0.05 versus Scr inhibitor). All data were from at least three independent experiments.

Figure S10: miR-578 mimic transfection affects endothelial cell function

RF/6A cells were transfected with Scr mimic, miR-578 mimic or left untreated (Ctrl). qRT-PCRs were conducted to detect the expression of FGF2, PDGF, NOTCH1, CXCR4, and MMP9. All data were from at least three independent experiments.

Figure S11: VEGFA/VCAM1 overexpression rescues cZBTB44 silencing-mediated effects on endothelial cell function

(A) RF/6A cells were treated as shown. Migration of RF/6A cells was measured using transwell assay and quantification of migrated cells was performed (n=4, **P*<0.05 versus Scr siRNA, #*P*<0.05 versus cZBTB44 siRNA + Vector). (B) RF/6A cells were seeded on the matrigel matrix. The tube-like structures were observed 4 h after cell seeding. Average length of tube formation for each field was statistically analyzed (n=4, **P*<0.05 versus Scr siRNA, #*P*<0.05 versus cZBTB44 siRNA + Vector). All data were from at least three independent experiments.

Figure S12: Detection of cZBTB44 expression in the plasma obtained from the patients with nAMD, ARC and glaucoma

qRT-PCRs were conducted to detect cZBTB44 expression in the EDTA-plasma obtained from the patients with nAMD, ARC, and glaucoma (n=15). All data were from at least three independent experiments.

Fig.S1

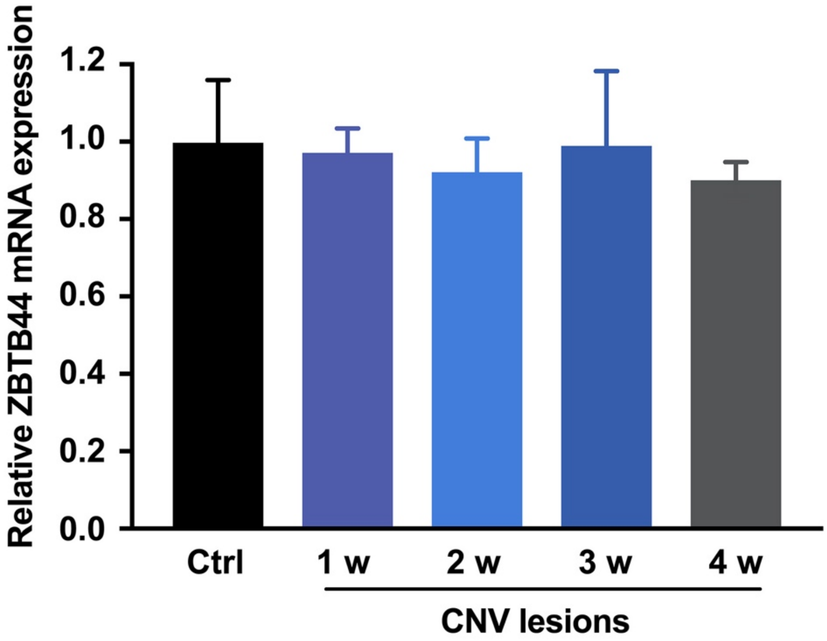
Relative ZBTB44 mRNA expression
1.2
1.0
0.8
0.6
0.4
0.2
0.0
Ctrl
1 w
2 w
3 w
4 w
CNV lesions

Fig.S2

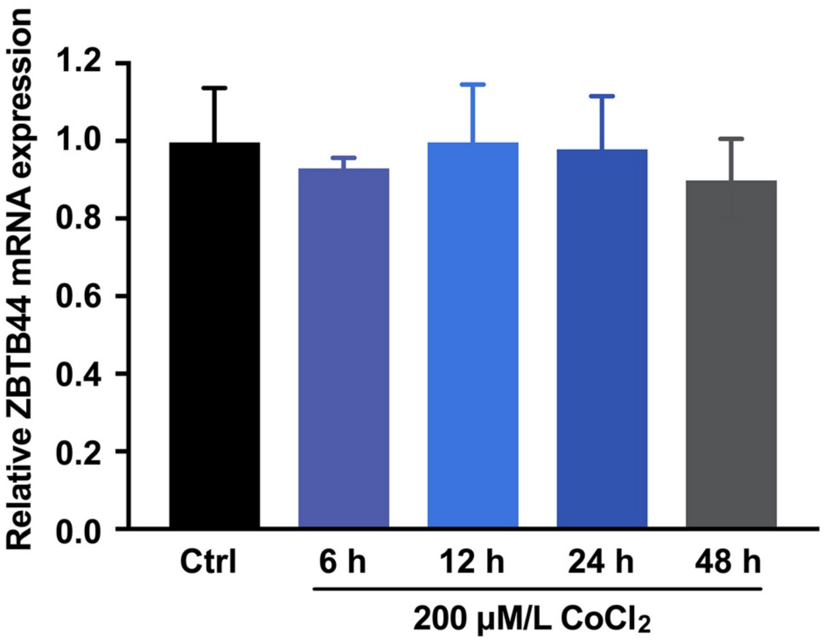

Relative ZBTB44 mRNA expression
1.2
1.0
0.8
0.6
0.4
0.2
0.0
Ctrl
6 h
12 h
24 h
48 h
200 μM/L CoCl2

Fig.S3

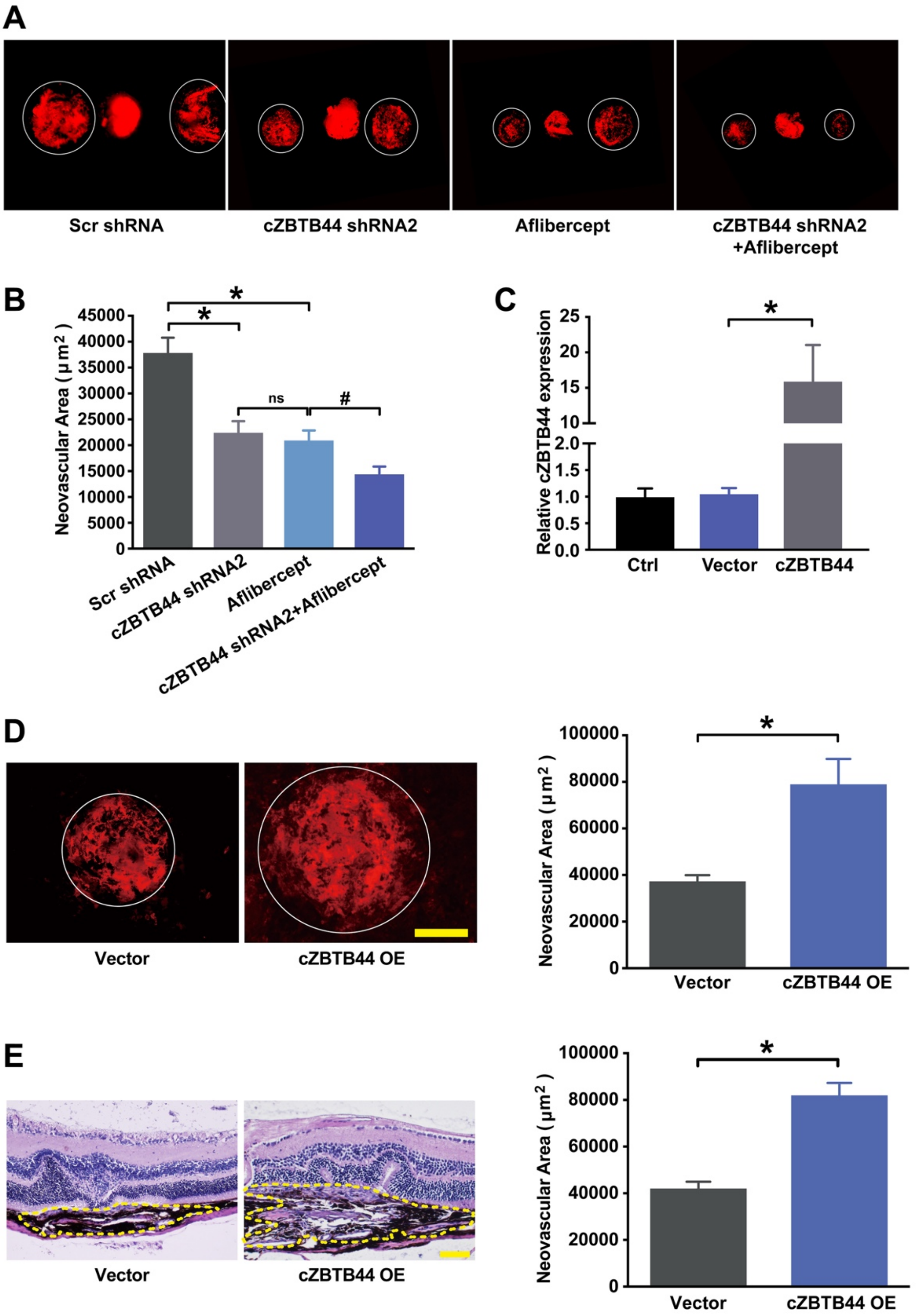
A
Scr shRNA
cZBTB44 shRNA2
Aflibercept
cZBTB44 shRNA2
+Aflibercept
B
Neovascular Area (μm²)
45000
40000
35000
30000
25000
20000
15000
10000
5000
0
*
*
ns
#
Scr shRNA
cZBTB44 shRNA2
Aflibercept
cZBTB44 shRNA2+Aflibercept
C
Relative cZBTB44 expression
25
20
15
10
2.0
1.5
1.0
0.5
0.0
*
Ctrl
Vector
cZBTB44
D
Vector
cZBTB44 OE
Neovascular Area (μm²)
100000
80000
60000
40000
20000
0
*
Vector
cZBTB44 OE
E
Vector
cZBTB44 OE
Neovascular Area (μm²)
100000
80000
60000
40000
20000
0
*
Vector
cZBTB44 OE

Fig.S4

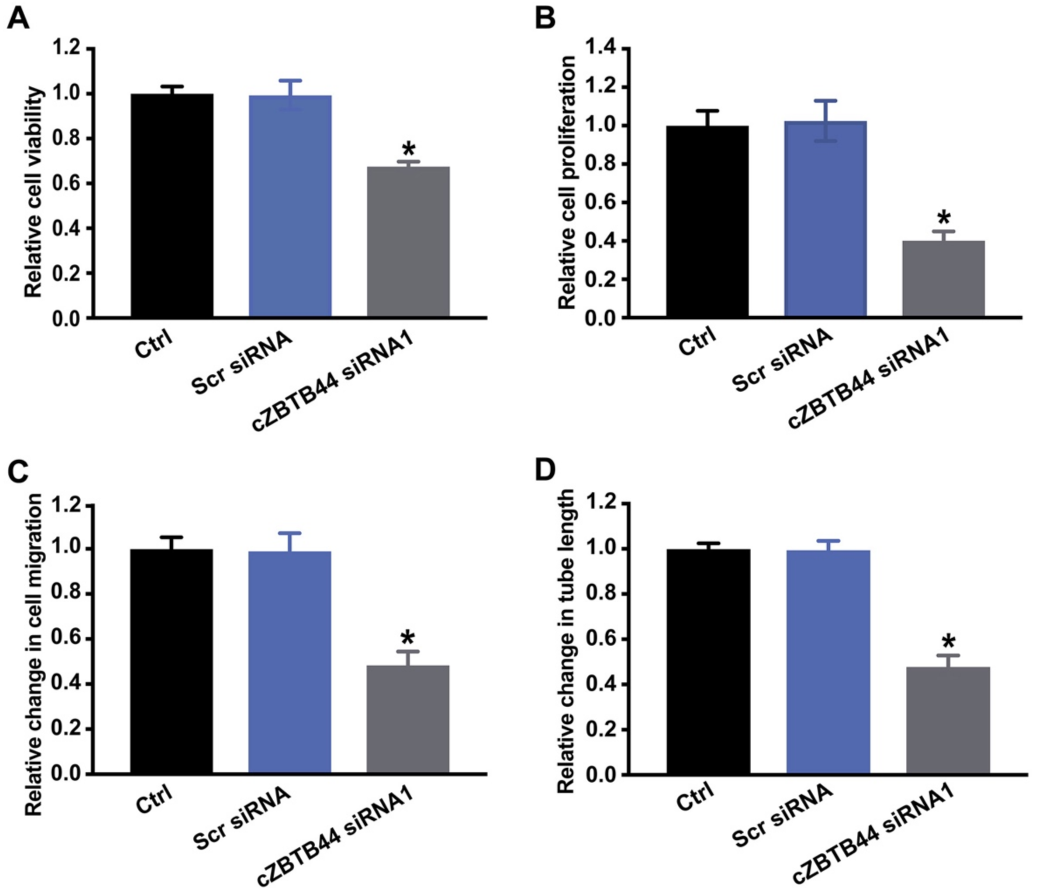

A
Relative cell viability
1.2
1.0
0.8
0.6
0.4
0.2
0.0
*
Ctrl
Scr siRNA
cZBTB44 siRNA1
B
Relative cell proliferation
1.4
1.2
1.0
0.8
0.6
0.4
0.2
0.0
*
Ctrl
Scr siRNA
cZBTB44 siRNA1
C
Relative change in cell migration
1.2
1.0
0.8
0.6
0.4
0.2
0.0
*
Ctrl
Scr siRNA
cZBTB44 siRNA1
D
Relative change in tube length
1.2
1.0
0.8
0.6
0.4
0.2
0.0
*
Ctrl
Scr siRNA
cZBTB44 siRNA1

Fig.S5

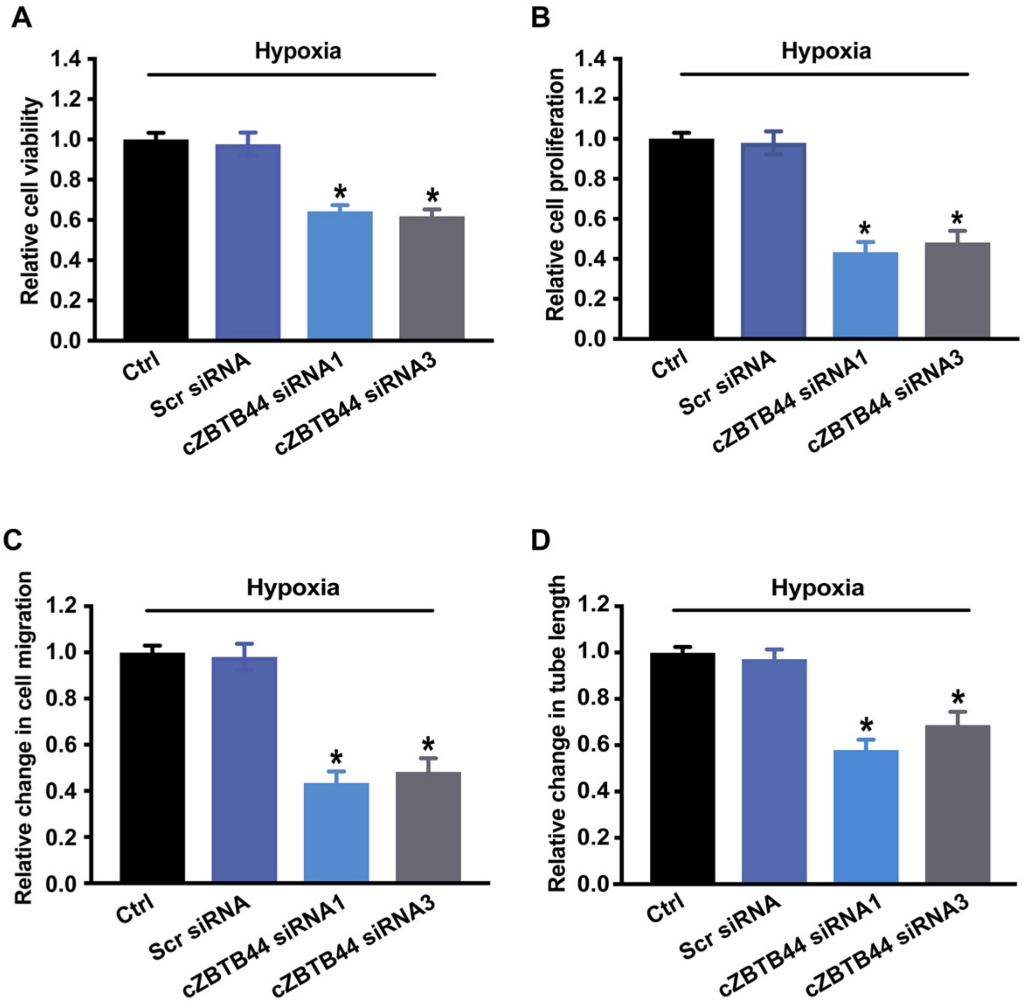
A
Hypoxia
Relative cell viability
1.4
1.2
1.0
0.8
0.6
0.4
0.2
0.0
Ctrl
Scr siRNA
cZBTB44 siRNA1
cZBTB44 siRNA3
*
*
B
Hypoxia
Relative cell proliferation
1.4
1.2
1.0
0.8
0.6
0.4
0.2
0.0
Ctrl
Scr siRNA
cZBTB44 siRNA1
cZBTB44 siRNA3
*
*
C
Hypoxia
Relative change in cell migration
1.2
1.0
0.8
0.6
0.4
0.2
0.0
Ctrl
Scr siRNA
cZBTB44 siRNA1
cZBTB44 siRNA3
*
*
D
Hypoxia
Relative change in tube length
1.2
1.0
0.8
0.6
0.4
0.2
0.0
Ctrl
Scr siRNA
cZBTB44 siRNA1
cZBTB44 siRNA3
*
*

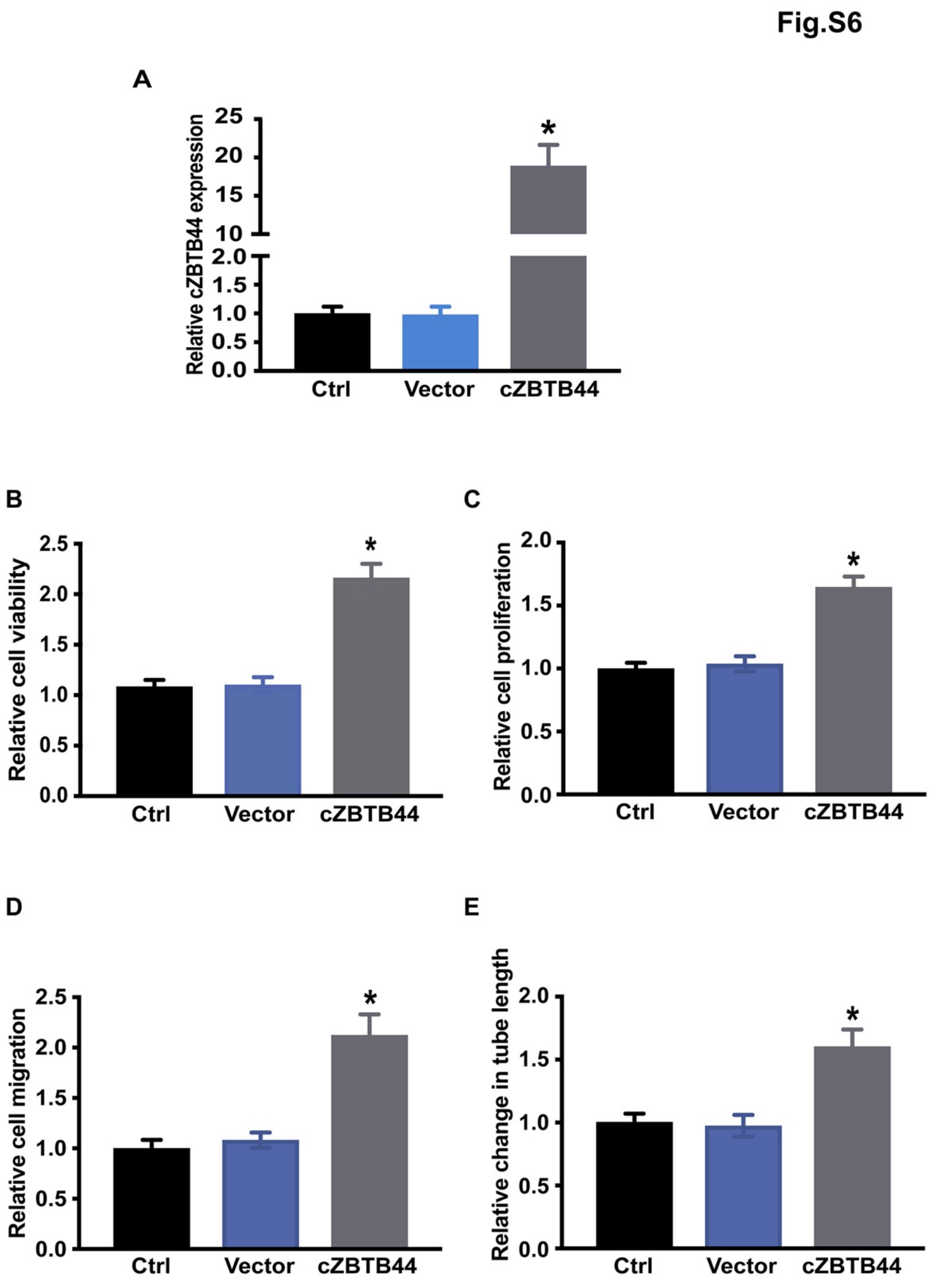
Fig.S6
A
Relative cZBTB44 expression
25
20
15
10
2.0
1.5
1.0
0.5
0.0
*
Ctrl
Vector
cZBTB44
B
Relative cell viability
2.5
2.0
1.5
1.0
0.5
0.0
*
Ctrl
Vector
cZBTB44
C
Relative cell proliferation
2.0
1.5
1.0
0.5
0.0
*
Ctrl
Vector
cZBTB44
D
Relative cell migration
2.5
2.0
1.5
1.0
0.5
0.0
*
Ctrl
Vector
cZBTB44
E
Relative change in tube length
2.0
1.5
1.0
0.5
0.0
*
Ctrl
Vector
cZBTB44

Fig.S7

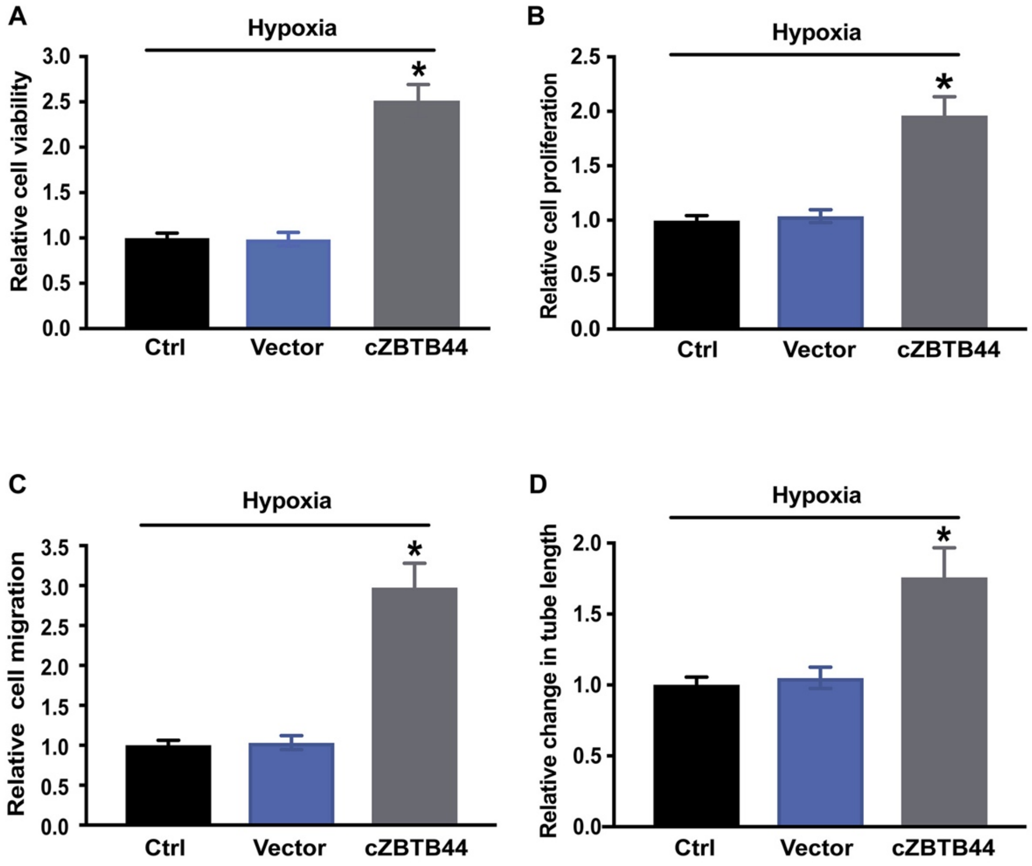

A
Hypoxia
Relative cell viability
3.0
2.5
2.0
1.5
1.0
0.5
0.0
*
Ctrl
Vector
cZBTB44
B
Hypoxia
Relative cell proliferation
2.5
2.0
1.5
1.0
0.5
0.0
*
Ctrl
Vector
cZBTB44
C
Hypoxia
Relative cell migration
3.5
3.0
2.5
2.0
1.5
1.0
0.5
0.0
*
Ctrl
Vector
cZBTB44
D
Hypoxia
Relative change in tube length
2.0
1.5
1.0
0.5
0.0
*
Ctrl
Vector
cZBTB44

Fig.S8

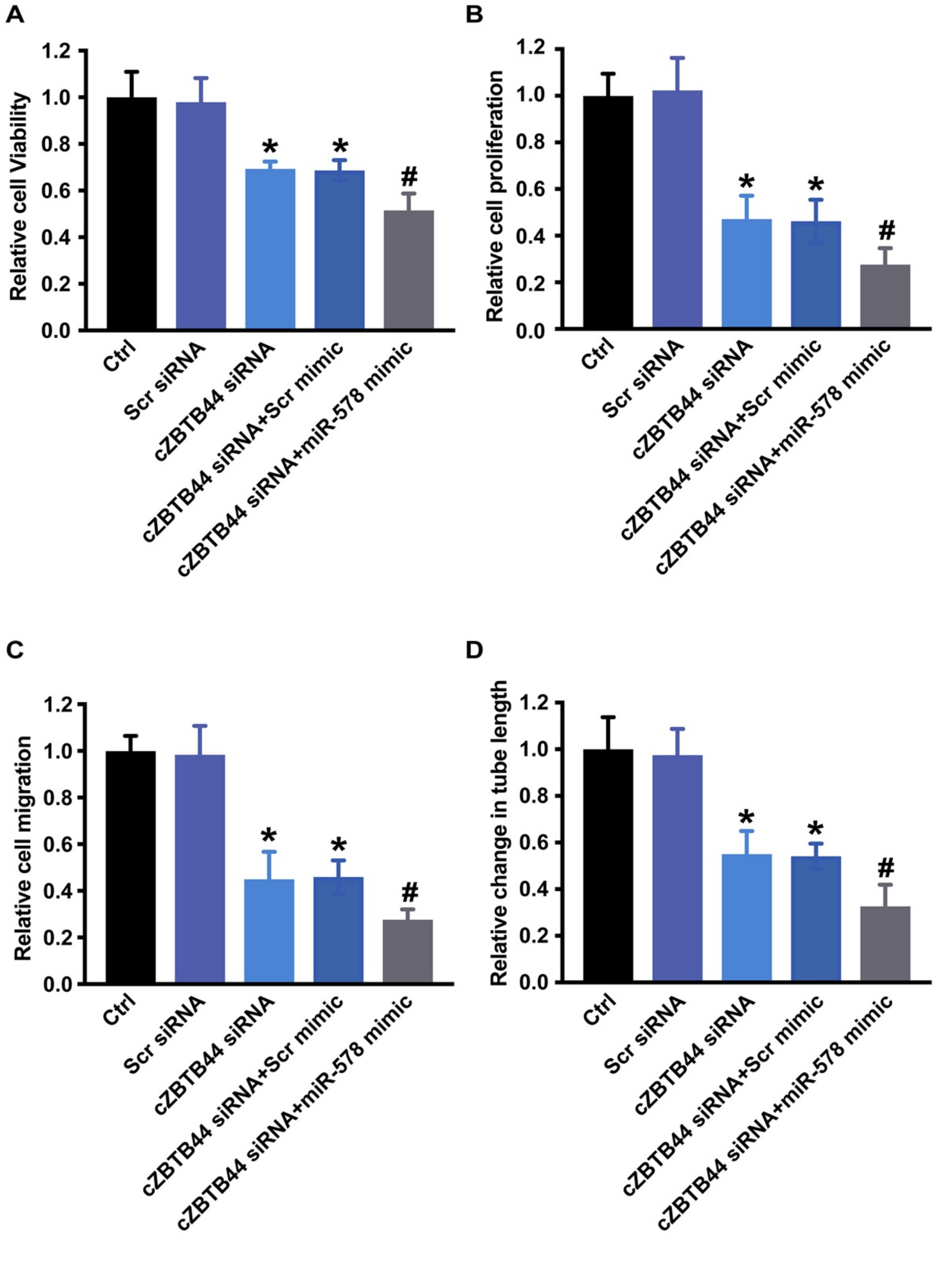
A
Relative cell Viability
1.2
1.0
0.8
0.6
0.4
0.2
0.0
Ctrl
Scr siRNA
cZBTB44 siRNA
cZBTB44 siRNA+Scr mimic
cZBTB44 siRNA+miR-578 mimic
B
Relative cell proliferation
C
Relative cell migration
D
Relative change in tube length

Fig.S9

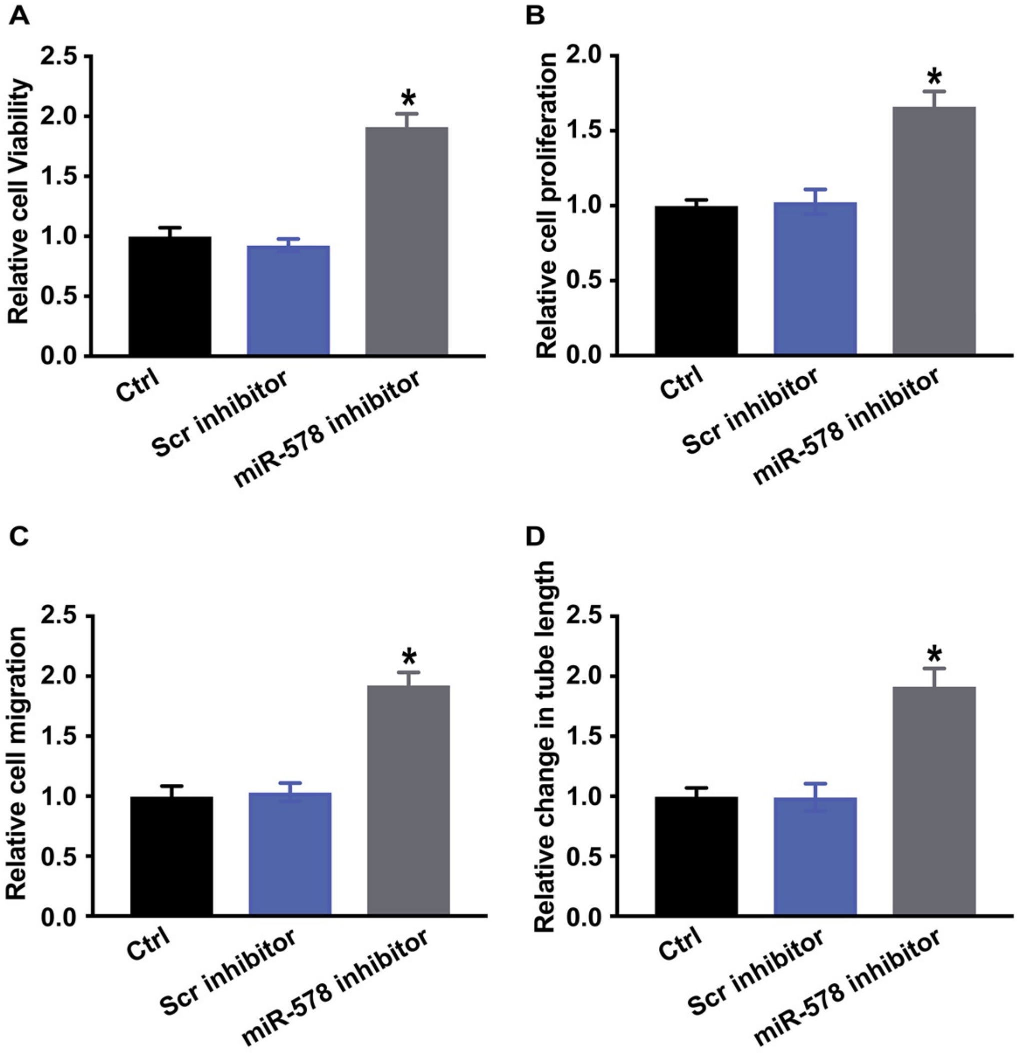
A
Relative cell Viability
2.5
2.0
1.5
1.0
0.5
0.0
*
Ctrl
Scr inhibitor
miR-578 inhibitor
B
Relative cell proliferation
2.0
1.5
1.0
0.5
0.0
*
Ctrl
Scr inhibitor
miR-578 inhibitor
C
Relative cell migration
2.5
2.0
1.5
1.0
0.5
0.0
*
Ctrl
Scr inhibitor
miR-578 inhibitor
D
Relative change in tube length
2.5
2.0
1.5
1.0
0.5
0.0
*
Ctrl
Scr inhibitor
miR-578 inhibitor

Fig.S10

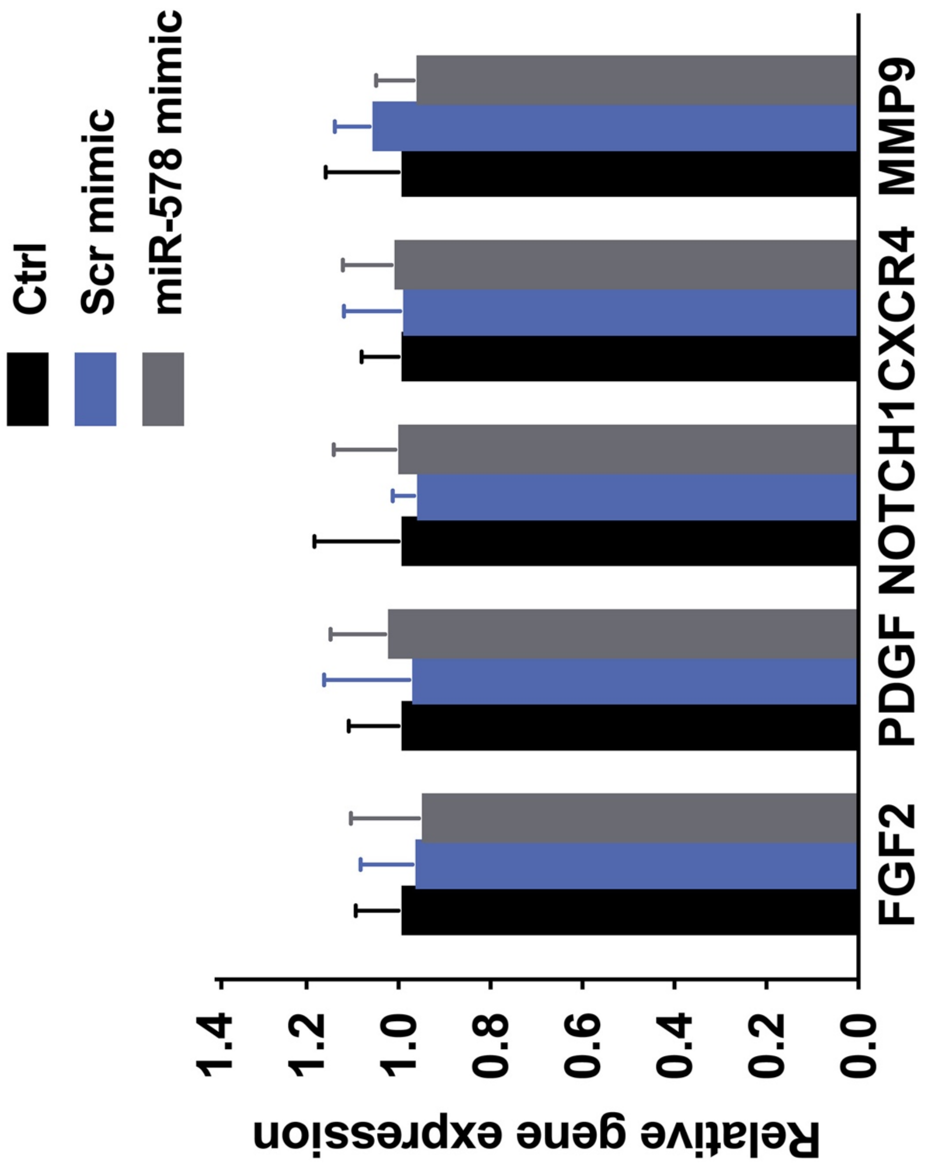
Ctrl
Scr mimic
miR-578 mimic
Relative gene expression
0.0
0.2
0.4
0.6
0.8
1.0
1.2
1.4
FGF2
PDGF
NOTCH1
CXCR4
MMP9

Fig.S11

A

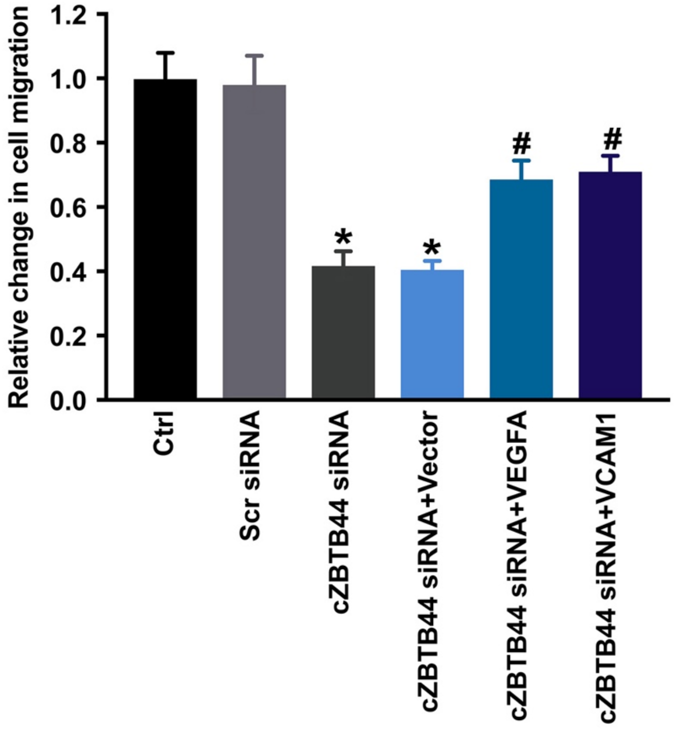

Relative change in cell migration
1.2
1.0
0.8
0.6
0.4
0.2
0.0
*
*
#
#
Ctrl
Scr siRNA
cZBTB44 siRNA
cZBTB44 siRNA+Vector
cZBTB44 siRNA+VEGFA
cZBTB44 siRNA+VCAM1

B

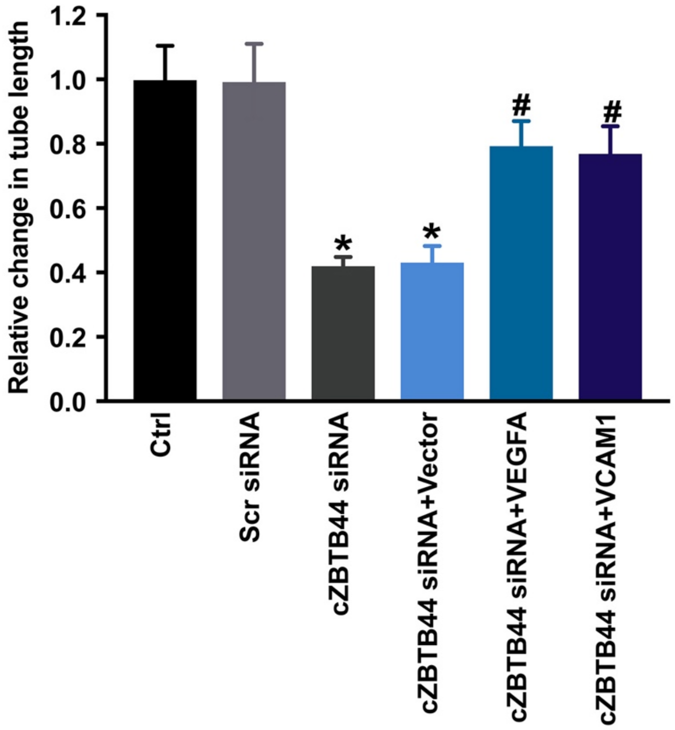

Relative change in tube length
1.2
1.0
0.8
0.6
0.4
0.2
0.0
*
*
#
#
Ctrl
Scr siRNA
cZBTB44 siRNA
cZBTB44 siRNA+Vector
cZBTB44 siRNA+VEGFA
cZBTB44 siRNA+VCAM1

Fig.S12

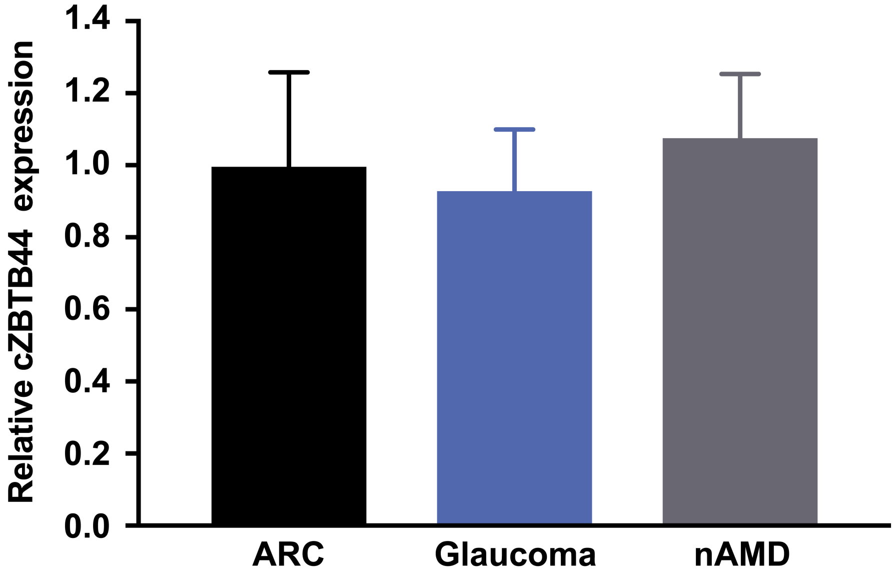

1.4
1.2
1.0
0.8
0.6
0.4
0.2
0.0
Relative cZBTB44 expression
ARC
Glaucoma
nAMD

Table S1: Sequences of qRT-PCR Primers

Gene Symbol	Primer	(5'-3')
**circZBTB44**	Forward	AAGTGTCCCAGCCTGTCAGT
	Reverse	AGCTTTCCAAGCATTTCCTG
**h-ZBTB44**	Forward	GAAGAAGATGTCCGGGTCAA
	Reverse	AGTGTAGGCAAGCCCTCAGA
**h-VCAM1**	Forward	TACAACCGTCTTGGTCAGCC
	Reverse	CCACAGGATTTTCGGAGCA
**h-VEGFA**	Forward	CCTCTTGGAATTGGATTCGC
	Reverse	TGTATGTGGGTGGGTGTGTCTAC
**h-FGF2**	Forward	GGGACTGGTCAGTATTAGAGGT
	Reverse	CTCTTGGAGTTCCGTCTTTGTT
**h-NOTCH1**	Forward	GAGGCGTGGCAGACTATGC
	Reverse	CTTGTACTCCGTCAGCGTGA
**h-CXCR4**	Forward	ACTACACCGAGGAAATGGGCT
	Reverse	CCCACAATGCCAGTTAAGAAGA
**h-PDGF**	Forward	GCTCCCGCAGACGTTTTTAC
	Reverse	CGAGAGACAGAGGTTTCGGG
**h-MMP9**	Forward	TTTAGCAAACGTAGGGGCGG
	Reverse	CTTCACGTCGAACCTGCGG
**h-GAPDH**	Forward	GCAACTAGGATGGTGTGGCT
	Reverse	TCCCATTCCCCAGCTCTCATA
**m-Vcam1**	Forward	CCAGACATTTACCCAGTTTACAGG
	Reverse	TGACGGGAGTAAAGGTTACTTCC
**m-Zbtb44**	Forward	ATCCGCAAGCCTCGGATTTA
	Reverse	AGAGTATGCGCAGTGGCAAT
**m-Vegfa**	Forward	TGGCTTACCCTTCCTCATCTT
	Reverse	TTCTCTTTTCTCTGCCTCCGT
**m-Gapdh**	Forward	TGAAATGTGCACGCACCAAG
	Reverse	GGGAAGCAGCATTCAGGTCT

Table S2: The clinic pathologic characteristics of patients used for plasma and aqueous homor collection

Patient No.	Age (years)	Sex	Amount of Total RNA (ng)		Relative cZBTB44 expression (Compared with ARC) ($\Delta\Delta C_T$)	
			Plasma	AH	Plasma	AH
ARC 1	69	M	1204.3	239	-0.19	0.30
ARC 2	74	M	1591	194.8	-0.05	-0.26
ARC 3	61	M	992.7	348.9	-0.04	-0.52
ARC 4	50	M	1130	356.1	0.21	0.35
ARC 5	61	M	955.7	237.5	0.20	0.48
ARC 6	63	F	1485	164.3	1.03	0.14
ARC 7	65	M	1332.5	172.7	0.79	-0.36
ARC 8	54	M	1013.9	315.1	-0.36	-0.70
ARC 9	65	F	1213.4	246.6	-0.12	0.37
ARC 10	70	F	1146.5	228.3	0.27	-0.05
ARC 11	57	F	1852.3	178	0.57	-0.01
ARC 12	59	F	985.4	318.6	0.07	0.32
ARC 13	53	M	1109	287.9	-0.18	-0.13
ARC 14	45	M	1245.9	270.6	-0.23	-0.19
ARC 15	63	M	996.2	168	0.38	0.21
Glaucoma 1	63	F	1372	360.6	-0.57	0.17
Glaucoma 2	66	M	1724.7	268.5	-0.07	0.40
Glaucoma 3	75	M	517.4	373.1	0.28	0.24
Glaucoma 4	53	F	1020	262.2	-0.73	0.34
Glaucoma 5	78	F	1175.1	307	-0.18	-0.18
Glaucoma 6	42	F	981	454.6	0.67	0.19
Glaucoma 7	51	F	602.2	291.3	0.27	0.03
Glaucoma 8	50	F	1399	372.3	-0.41	-0.36
Glaucoma 9	59	M	591.5	383.8	-0.87	0.25
Glaucoma 10	66	F	974.1	580.3	0.44	-0.19
Glaucoma 11	43	F	989	382.8	0.07	0.22
Glaucoma 12	65	F	906.1	267.3	0.58	-0.35
Glaucoma 13	46	M	825.1	448.5	-0.27	-0.07
Glaucoma 14	61	M	1021.3	188.9	0.36	0.18
Glaucoma 15	66	M	683.4	191.7	0.41	0.25
nAMD 1	55	F	537.6	389.7	-1.89	-0.31
nAMD 2	63	F	1089.3	336.2	-0.45	-0.05
nAMD 3	61	F	519	251.6	-2.04	-0.08
nAMD 4	64	M	1351.8	280.6	-1.21	-0.46
nAMD 5	50	F	1120	312	-0.75	-0.27
nAMD 6	46	F	710.1	215.4	-1.03	0.16
nAMD 7	48	M	611.1	320.8	-0.79	-0.14
nAMD 8	56	F	1018	372.9	-1.36	-0.19

nAMD 9	59	F	743	184.7	-0.92	-0.26
nAMD 10	52	M	1283.6	162.2	-1.77	-0.02
nAMD 11	60	F	703.7	319.1	-0.57	0.23
nAMD 12	59	F	1042.3	211.2	-1.07	-0.58
nAMD 13	59	F	788.2	298.2	-2.01	-0.28
nAMD 14	62	F	575.6	283.6	-0.73	0.15
nAMD 15	41	F	957.2	421.5	-2.06	0.03

Note: The patients with diabetes mellitus, cardiovascular diseases and other systemic diseases were excluded.
